# Plastid-mediated feedback regulation of Arabidopsis *LYCOPENE EPSILON CYCLASE* is modulated by the promoter and a 5′UTR structural variant harbouring a conserved IRES

**DOI:** 10.1186/s12915-025-02436-z

**Published:** 2025-11-04

**Authors:** Yagiz Alagoz, Jwalit J. Nayak, Rishi Aryal, Jacinta L. Watkins, Sophie Holland, David T. Tissue, Barry J. Pogson, Christopher I. Cazzonelli

**Affiliations:** 1 Hawkesbury Institute for the Environment, Western Sydney University, Locked Bag 1797, Penrith, NSW 2751 Australia; 2 ARC Training Centre for Accelerated Future Crops Development, Research School of Biology, College of Science, The Australian National University, Canberra, ACT 2601 Australia; 3 Present address: Center of Excellence for Sustainable Food Security, King Abdullah University of Science and Technology (KAUST), Thuwal, 23955-6900 Kingdom of Saudi Arabia; 4 Present address: Department of Horticultural Science, North Carolina State University, Raleigh, NC 27695 USA; 5 Present address: Centre for Agriculture and the Bioeconomy, Queensland University of Technology, Brisbane, QLD 4001 Australia

**Keywords:** 5′UTR, *LYCOPENE EPSILON CYCLASE*, Apocarotenoid signal, RNA regulation, Structural switch, IRES, Expression platform, Aptamer domain, Metabolic feedback, Carotenoid, Promoter, Riboswitch, Retrograde, *Arabidopsis*

## Abstract

**Background:**

Perturbations in flux through the carotenoid pathway can signal control over nuclear gene expression to maintain metabolic homeostasis in plastids; however, the genetic targets and mechanisms are rarely characterised in plants.

**Results:**

Utilising mutants, over- and exogenous expression lines, and chemical inhibitors of carotenogenesis, we demonstrate that *Arabidopsis LYCOPENE EPSILON CYCLASE* (*εLCY*) mRNA levels correlate with changes in the accumulation of cyclic carotenoids generated by the β-branch in the metabolic pathway.

In dark- and light-grown seedlings subjected to retrograde feedback signalling triggered by norflurazon and the loss-of-function in CAROTENOID ISOMERASE (CRTISO) activity, changes in *εLCY* levels were mirrored by *FIREFLY LUCIFERASE* (*FiLUC*) levels regulated by the *εLCY* promoter in isogenic transgenic seedlings. The *εLCY* promoter enabled three alternative transcription start sites (TSS), and the most prominent 5′UTR fragment generated in dark- and light-grown seedlings harboured a 5′ conserved domain (CD) with other *Brassicaceae* species adjacent to a conserved viral internal ribosome entry site (IRES) sequence proximal to the start codon. In silico modelling predicted the 5′UTR formed two energetically separated RNA structural probabilities distinguished by alternative hairpin structures within the CD that lacked a centroid structure, unlike the adjacent IRES sequence that displayed a prominent centroid peak, higher base-pairing probability, and lower positional entropy indicative of fewer secondary structure outcomes. Unlike the native 5′UTR, site-specific mutations predicted to stabilize the 5′UTR shape triggered post-transcriptional repression of FiLUC activity enabled by the *CaMV35S* promoter in transient tobacco and stable transgenic *Arabidopsis* tissues. The mutated 5′UTR RNA shape fragment conferred *CaMV35S* enabled reporter responsiveness to feedback signalling in *crtiso* mutant and wild type (WT) etiolated and de-etiolated seedlings treated with norflurazon.

**Conclusions:**

We propose that the *εLCY* promoter and 5′UTR harbour an expression platform, whereby alternative TSS and structural definitions enable transcriptional and post-transcriptional regulation in response to feedback signalling during plastid biogenesis and plant development.

**Supplementary Information:**

The online version contains supplementary material available at 10.1186/s12915-025-02436-z.

## One-sentence summary

The *LYCOPENE EPSILON CYCLASE* promoter including the 5′ untranslated leader region modulates responsiveness to perturbations in carotenoid signalling.

## Background

Metabolic feedback regulation is a cellular process whereby changes in the product of a biochemical pathway influences biosynthesis by altering gene expression, protein levels, and/or enzyme activity through allosteric alterations to balance pathway homeostasis within and between energy organelles and cells. This self-regulating mechanism requires a signal that can positively or negatively feedback or forward within their own biosynthetic pathway or influence upstream/downstream metabolic pathways to amplify or dampen the response respectively, ensuring the finely tuning of metabolism and cellular acclimation to internal or external environment changes [1, 2]. Carotenoids are secondary metabolites having a 40-carbon backbone that serve as pigments contributing to photosynthesis, photoprotection, and are substrates for the generation of apocarotenoid signalling metabolites, as well as phytohormones [3, 4]. Carotenoid biosynthesis, accumulation, and catabolism in plastids can be controlled by feedback signalling, a process potentially involving metabolites, phytohormones, miRNA, and/or proteins, which can be induced by genetic and chemical perturbations, as well as environmental change (e.g., light, temperature, and water stress), and developmental transitions [5–7].

Carotenoids are precursors of some phytohormones such as abscisic acid (ABA) and strigolactones (SL) as well as various apocarotenoids [8, 9]. Transcriptional responses associated with any carotenoid cleavage product, herein referred to as an apocarotenoid signal (ACS), have been linked to the control over carotenoid metabolic flux, plastid biogenesis and differentiation, leaf and root development, photo-acclimation, plant root-mycorrhizal interactions, herbivore defence, and plant growth [10–15]. A yet-to-be-discovered linear *cis*-carotene-derived retrograde apocarotenoid signal (*cis*-ACS) generated in plastids was shown to transcriptionally regulate key repressor (e.g., PHYTOCHROME INTERACTING FACTOR3; PIF3) and activator (e.g., ELONGATED HYPOCOTYL5; HY5) transcription factors that modulate *PHOTOSYNTHESIS ASSOCIATED NUCLEAR GENES* (*PhANGs*) and their protein products required to differentiate etioplasts into chloroplasts during the dark to light transition and hence promote photosynthesis [10, 12]. *PhANG* expression can also be suppressed in seedlings treated with norflurazon (NFZ) that blocks carotenoid biosynthesis and generates a plastid-derived retrograde signal that communicates with the nucleus to modulate plastid biogenesis [16]. Establishing the carotenoid substrate(s) and at what threshold level they could trigger retrograde feedback regulation of *PhANG* expression can be a challenge since the substrate(s) concentration is more abundant than normally required to produce a bioactive signal.

Feedback regulation due to metabolite signals could involve an interaction between a mobile bioactive metabolic signal and ligand-binding sensory domain (messenger RNA, DNA promoter *cis*-acting element, and/or transcription factor/enzyme active site) that causes aptamer alterations in nucleotide sequence structure, DNA–protein binding, or protein–protein interactions, leading to changes in transcriptional and/or translational expression platforms that modulate metabolite biosynthesis [17–19]. Molecular models have proposed how carotenoid derived signals could block enzyme catalysis, alter intron splicing, or affect protein–protein interactions [20–22]. The genetic targets amenable to carotenoid feedback signalling and mechanisms modulating transcription, and/or post-transcriptional processes remain to be elucidated.

LYCOPENE EPSILON CYCLASE (εLCY) regulates the α-branch of the carotenoid biosynthetic pathway, rate-limiting production of lutein (Figure S1), the most abundant carotenoid in foliar tissues [23, 24]. Intriguingly, modulation of precursor synthesis through the alpha- and beta-branches of the pathway changes flux and *eLCY* transcript levels (Additional file 1: Table S1; Figure S1). We hypothesized that changes in β-branch carotenoids might signal nuclear control over *εLCY* expression to balance pathway flux. For example, the exogenous expression of the *Pantoea ananatis PHYTOENE DESATURASE* (*CRTI*) gene in wild-type tomato, tangerine, and old gold crimson varieties enhanced β-carotenoids and *εLCY* expression during fruit ripening and the mechanism for this change in flux remains unclear [25] (Additional file 2: Table S1; Figure S1). Similarly, dark-grown *Arabidopsis ccr2* (*carotenoid* c*hloroplast regulator 2*) seedlings that lack function of CAROTENOID ISOMERASE (CRTISO) activity do not biosynthesize cyclic carotenoids (closed carbon ring structures at the ends), accumulate acyclic *cis*-carotenes (open linear chain of carbons without any ring structure), and exhibit a negative regulation in *εLCY* expression [26, 27] (Additional file 1: Figure S1). During *ccr2* seedling de-etiolation cyclic carotenoids begin to accumulate as light facilitates photoisomerization of tetra-*cis*-lycopene (prolycopene) into all-*trans*-lycopene (lycopene). *εLCY* expression was also reduced in older mature rosette leaves from the *ccr1* (*carotenoid* c*hloroplast regulator 1*) mutant that lacks function in SET DOMAIN GROUP 8 (SDG8) required to maintain *CRTISO* expression [28]. The changes in plastid generated acyclic *cis*-carotenes and/or cyclic carotenoids that correlate with genetic, environmental, and developmental regulation of *εLCY* expression in the nucleus have yet to be defined.

Manipulation of *εLCY* expression or activity has been adopted as a biofortification strategy to enhance β-carotene (pro-vitamin A) accumulation in crops [29–34]. εLCY opposes the production of β-branch carotenoids [35]. The downregulation of *εLCY* gene expression by genetic modifications or loss-of-function of εLCY activity redirects flux towards the β-branch in the pathway (10 out 14 published reports; Additional file 2: Table S1; Additional file 1: Figure S1). For example, transposon insertions and allelic variation in the 5′ upstream regions adjacent to the *εLCY* mRNA start codon can determine flux between the lutein and β-carotene branches, leading to β-carotene accumulation in maize and wheat [36–38]. Deciphering upstream regulatory components that modulate *εLCY* expression could reveal mechanism by which feedback signalling modulates the branch in carotenoid biosynthesis as well as elucidate the potential source of the signal.

The upstream promoter and 5′ untranslated region (UTR) can coordinate transcriptional and post-transcriptional regulation. Changes in the transcription start site (TSS) can modify the 5′UTR length, splicing, presence/absence of regulatory motifs, and RNA structural formation that impact gene transcription, mRNA decay, and/or protein translation [39]. For example, in *Arabidopsis*, the *PHYTOENE SYNTHASE* (*PSY*) 5′UTR controlled carotenogenesis by altering the abundance of *PSY* splice variants during different light conditions [20]. Changes to RNA shape definition in a temperature-dependent manner (e.g., RNA thermoswitches) can regulate RNA stability, pre-mRNA splicing, and/or translation efficiency [40, 41]. In fact, some UTRs from prokaryote genes can harbour RNA structural domains that sense/bind metabolites (e.g., metabolite-binding RNA riboswitches) and one was conserved in plants (e.g. *THIAMINE PYROPHOSPHATE; TPP* riboswitch) [42–44]. Other 5′UTR structural definitions such as the Internal Ribosome Entry Site (IRES) modulate translation initiation and environmental responses by exploiting RNA structure flexibility, and therefore plasticity, as a core functional element [45–48]. While IRES elements are common to plant viruses, a small number have been identified in vertebrate, insect and yeast, and rarely in endogenous plant cellular messenger RNAs [49]. The presence of a short upstream open reading frame (uORF) in the 5′UTR can also contribute to the regulation of translation in plants and cause RNA remodelling affecting the formation of an active IRES [50, 51]. Such upstream RNA components are hallmark features for regulating metabolic and environmental feedback signalling and have yet to be linked to the control over carotenoid homeostasis *in planta*.

Here, we utilised a genetic (*crtiso* mutant; *ccr2*), environmental (etiolated vs de-etiolated seedlings), chemical (norflurazon; NFZ, blocks carotenoid biosynthesis and signals changes in nuclear gene expression), and developmental changes (young emerging vs mature leaves) to alter individual carotenoid levels and correlated feedback signalling with *εLCY* expression *in planta*. We engineered promoter-reporter gene fusions and utilised stable transgenics as well as transient expression systems to unravel mechanisms by which an exogenously expressed *εLCY* promoter-luciferase transgene and endogenous *εLCY* mRNA levels are co-regulated in tissues enriched in etioplasts (dark) or chloroplasts (light). Transcription initiation sites were identified within the tested *εLCY* promoter fragment. In silico analysis identified conserved domains and a structural motif, prompting a hypothesis that the mRNA 5' untranslated leader region (5′UTR) could function to modulate promoter activity in response to carotenoid feedback signalling. Bioinformatics modelling predicted alternative 5′UTR RNA structural definitions, and mutational/gain-of-function promoter-reporter assays *in planta* were utilised to test the hypothesis. We describe a peculiar RNA structural definition within the most abundant *εLCY* 5′UTR fragment that appears to modulate FiLUC reporter activity in response to retrograde feedback signalling triggered by *ccr2* and in NFZ treatment of seedlings, as well as during leaf development and photomorphogenesis.

## Results

### Linear *cis*-carotene-derived signalling does not trigger feedback regulation of *εLCY* expression

**Fig. 1:**
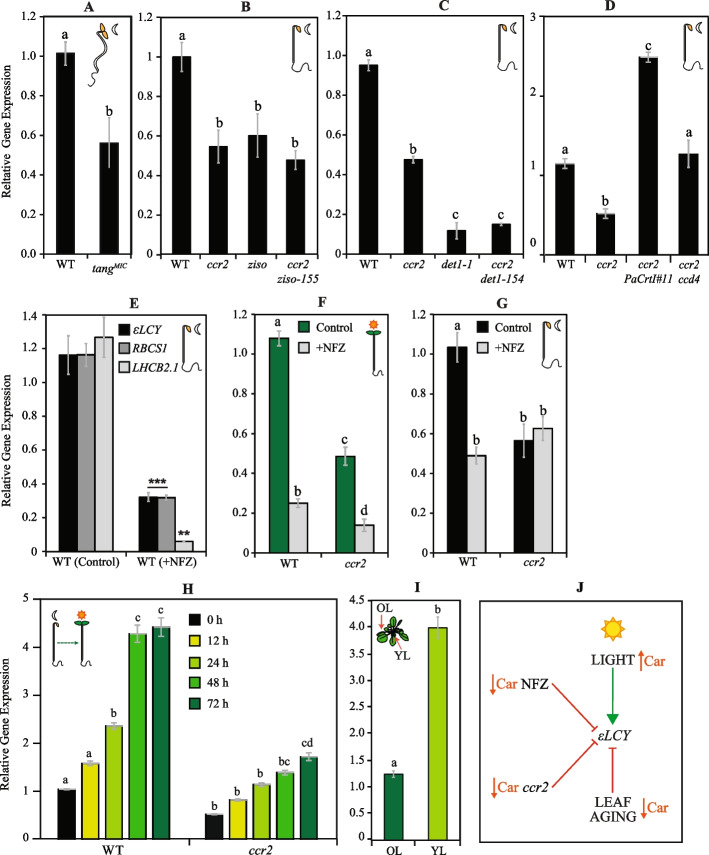
Regulation of *εLCY* expression by chemical, genetic, developmental, and environmental changes in seedling and leaf tissues. **A–C** *εLCY* expression in etiolated tissues from **A** MicroTom *tangerine* (*tang*.^*Mic*^), **B** *ccr2*, *ziso*, and *ccr2 ziso*, and **C** *det1-154*, *ccr2*, and *ccr2 det1-154* that can only accumulate linear *cis*-carotenes. **D** *εLCY* expression in WT, *ccr2*, *ccr2 T35ehn*::*PaCrtI#11* (*ccr2* transformed with pT35enh::SSU-*PaCrtI*), and *ccr2 ccd4* etiolated seedlings that accumulate linear *cis*-carotenes plus other cyclic carotenoids. **E** Relative expression of photosynthesis-associated nuclear genes (*RBCS1*, *LHCB2.1*) and *εLCY* in WT etiolated seedling tissues treated with norflurazon (+ NFZ) or water (Control). **F** *εLCY* expression in de-etiolated WT and *ccr2* seedling tissues treated with NFZ (+ NFZ) or water (Control).** G** *εLCY* expression in etiolated *ccr2* seedling tissues treated with NFZ (+ NFZ) or water (Control). **H** *εLCY* expression in WT and *ccr2* etiolated and de-etiolated seedlings exposed to continuous light for 12, 24, 48, and 72 h. Gene expression levels are relative to WT 0 h treatment. **I** *εLCY* expression in young emerging leaves (YL) relative to older mature leaves (OL) from whole rosettes grown in soil under a 16-h photoperiod. **J** Model summarising the regulation of *εLCY* expression triggered by chemical (NFZ), genetic (*ccr2*), developmental (leaf age), and environmental (light) perturbations. The up and down arrows in orange represent higher and lower β-carotenoid (Car) levels. Data is representative of two to three independent experiments, and standard error bars of the mean are displayed (*n* = 3–9). Lettering denotes significance by one- or two-way ANOVA statistical analysis using the post hoc Tukey test. Abbreviations: *εLCY*; *LYCOPENE EPSILON CYCLASE*, *RBCS1; RIBULOSE BISPHOSPHATE CARBOXYLASE SMALL CHAIN 1 A, LHCB2.1; LIGHT-HARVESTING CHLOROPHYLL B-BINDING 2, ccr2*; *carotenoid chloroplast regulator 2*

We utilised carotenoid biosynthetic mutants accumulating linear *cis*-carotenes in dark-grown seedlings to decipher whether they trigger feedback regulation of *εLCY* expression. The tomato Micro-Tom *tangerine* (*tang*^*Mic*^) and *Arabidopsis ccr2* mutants that have impaired CRTISO activity and accumulate linear *cis*-carotenes (mostly neurosporene and tetra-*cis*-lycopene) [26, 52] show reduced *εLCY* transcript levels in dark-grown seedlings (Fig. 1A,B). The loss-of-function of ZETA-CAROTENE ISOMERASE (*ziso*) in the *Arabidopsis ccr2 ziso* double mutant reduced total *cis*-carotene levels by 50% and prevented biosynthesis of key substrates, neurosporene and tetra-*cis*-lycopene (Additional file 1: Figure S2A), previously associated with a perturbation in prolamellar body (PLB) formation in *ccr2* [10]. *ccr2*, *ziso*, and *ccr2 ziso* showed a similar two-fold reduction in *εLCY* expression (Fig. 1B). The loss-of-function of DE-ETIOLATED 1 (DET1) in *ccr2 det1-154* dark-grown seedlings reduced di-*cis*-ζ-carotene, neurosporene, and tetra-*cis*-lycopene levels as well as restored PLB formation in *ccr2* [10]. *ccr2 det1-154* etiolated seedlings exhibit a similar 80% reduction in *εLCY* expression compared to *det1-1* (Fig. 1C). Therefore, since *ccr2 ziso* and *ccr2 det1-154* were previously shown to impair *cis*-carotene-derived signalling, restore plastid biogenesis and *PhANG* expression in *ccr2*, alterations in *cis*-carotene accumulation are unlikely to cause negative feedback regulation of *εLCY* expression in *ccr2* dark-grown seedlings.

### Increasing cyclic carotenoid levels correlates with enhanced *εLCY* transcript levels

We next queried if changes in cyclic carotenoids downstream of linear *cis*-carotene biosynthesis can feedback to regulate *εLCY* expression. *εLCY* expression was reduced by ~ 80% in *det1-1* etiolated seedlings (Fig. 1C) previously shown to have reduced (threefold) cyclic carotenoids compared to WT [10]. The biosynthesis of acyclic all-*trans*-lycopene (linear carbon double bonds in the trans-configuration) can bypass *cis*-carotene precursors in plants by exogenously expressing *Pantoea agglomerans* (*Erwinia herbicola*) or *Pantoea ananatis* (*Erwinia uredovora*) *CAROTENOID ISOMERASE* (*PaCrtI*) [25, 53, 54]. A transgene harbouring *PaCrtI* fused downstream of the *Arabidopsis* small subunit (SSU) of RuBisCo transit peptide (56 amino acids) [55] was placed under the control of a CaMV35S promoter (*T35enh*::*SSU-PaCrtI*) and transformed into *ccr2* to enhance all-*trans*-lycopene biosynthesis in etiolated *ccr2* seedlings. Homozygous *ccr2 35S::PaCrtI* lines (#5, 9, 11, and 26) that segregated in a typical 3:1 manner (Chi^2 *p*-value > 0.64) displayed a carotenoid composition similar to that of *ccr2* when grown under a long 16 h photoperiod (Additional file 2: Table S2). Etiolated tissues from *ccr2 T35enh::SSU-PaCrtI*#11 showed a 2.3- to 3.8-fold reduction in linear *cis*-carotene levels correlating with a two- to three-fold increase in neoxanthin, violaxanthin, antheraxanthin, and β-carotene (Additional file 1: Figure S2B-C). *ccr2 T35enh::SSU-PaCrtI*#11 etiolated seedlings displayed a five-fold and 2.4-fold higher *εLCY* transcript abundance compared to *ccr2* and WT, respectively (Fig. 1D). Therefore, the increase in cyclic carotenoids in etiolated *ccr2* tissues caused by exogenous expression of *PaCrtI* correlated with enhanced *εLCY* transcript levels.

CAROTENOID CLEAVAGE DIOXYGENASE 4 (CCD4) modulates β-carotene accumulation in *Arabidopsis* as *ccd4* mutants show enhanced β-carotene accumulation in seeds and senescing leaves [56–58]. We tested if *ccd4* introgressed with *ccr2* would enrich cyclic carotenoid levels and alter *εLCY* mRNA levels in etiolated tissues. Compared to *ccr2*, the *ccr2 ccd4* etiolated seedlings had a similar acyclic *cis*-carotene abundance yet significantly higher levels of β-carotene and zeaxanthin (Additional file 1: Figure S3A-B). *ccr2 ccd4* etiolated tissues showed a two-fold increase in *εLCY* expression relative to *ccr2* (Fig. 1D). Hence, the increased cyclic carotenoid accumulation in *ccr2 ccd4* correlated with enhanced *εLCY* expression.

### Chemical inhibition of cyclic carotenoid biosynthesis suppresses *εLCY* expression

Norflurazon (NFZ) chemically inhibits PDS enzyme activity and blocks carotenoid biosynthesis triggering a reduction in the expression of *PhANGs* (e.g., *LHCB2.1*) [59–61]. NFZ blocked the biosynthesis of cyclic carotenoids in WT etiolated seedlings, leaving only trace levels of lutein, zeaxanthin, and β-carotene discernible from residual seed-derived pigments (Additional file 1: Figure S4A). During skotomorphogenesis and photomorphogenesis, untreated (control) and NFZ-treated seedlings grew normally except that NFZ caused a bleached cotyledon phenotype (Additional file 1: Figure S4C). NFZ treatment of WT etiolated seedlings significantly reduced the expression of *LHCB2.1* (94%), *RBCS1* (65%), and *εLCY* (65%) relative to the untreated control (Fig. 1E). Similarly, NFZ treatment of WT de-etiolated seedlings caused a 4.3-fold reduction in *εLCY* expression (Fig. 1F). Therefore, NFZ-mediated inhibition of carotenoid biosynthesis (except phytoene and phytofluene) suppressed *εLCY* expression in etiolated and de-etiolated WT seedlings.

Etiolated *ccr2* seedlings cannot synthesise cyclic carotenoids and NFZ treatment did not affect *εLCY* transcript levels that were already lower compared to WT (Fig. 1G). NFZ treatment of etiolated *ccr2* seedlings blocked the synthesis of some *cis*-carotenes (e.g., ζ-carotene, neurosporene, and tetra-*cis*-lycopene), hyper-induced phytoene (fourfold like in WT), and reduced phytofluene (~ threefold) levels compared to untreated *ccr2* etiolated seedlings (Additional file 1: Figure S4B). The NFZ-induced changes in *cis*-carotene levels had no impact on *εLCY* expression. De-etiolation of *ccr2* seedlings allows photoisomerization of tetra-*cis*-lycopene into all-trans-lycopene and cyclic carotenoids accumulated to 50% of WT levels (Additional file 1: Figure S1, S5A). NFZ treatment further repressed *εLCY* expression (3.5-fold) in de-etiolated *ccr2* seedlings more so than in WT (Fig. 1F). Therefore, while NFZ does not affect *εLCY* expression in etiolated *ccr2* seedlings, it has an additive effect to further repress *εLCY* mRNA levels in de-etiolated *ccr2* seedlings indicating feedback signalling of *εLCY* was hyper-responsive to NFZ within the light environment.

### Light-enhanced *εLCY* mRNA levels are suppressed in de-etiolated *ccr2* seedlings

We investigated whether the expression of *εLCY* was altered during seedling photomorphogenesis and leaf development by comparing etiolated vs. de-etiolated cotyledon tissues and younger vs. fully expanded older leaf tissues, respectively, from WT and *ccr2*. Relative to etiolated seedlings, the expression of *εLCY* significantly increased after 24 h in de-etiolated WT seedlings and there was 4.5-fold higher *εLCY* expression 72 h after photomorphogenesis (Fig. 1H). Intriguingly, the expression of *εLCY* was consistently reduced (2- to 2.5-fold) at all time points in both etiolated and de-etiolated *ccr2* seedlings compared to WT (Fig. 1H). Cyclic carotenoids remained 50% lower in *ccr2* relative to WT de-etiolated seedlings (Additional file 1: Figure S5A). Therefore, carotenoids and *εLCY* transcript levels increase in WT and to a lesser extent in *ccr2* de-etiolated seedlings, during photomorphogenesis.

Bioinformatics analysis using the *Arabidopsis* TAIR eFP browser revealed that the absolute expression of *εLCY* was strongest in younger rosette leaves (Additional file 1: Figure S5B). We confirmed that *εLCY* transcript levels were four-fold higher in younger relative to older WT leaves (Fig. 1I). Young emerging leaves (leaf #10–13) contained higher carotenoid (and chlorophyll) levels compared to older fully expanded leaves (leaf #3–4) and do not normally accumulate linear *cis*-carotenes (Additional file 1: Figure S5C-D). Therefore, during leaf aging and development, there is a decline in cyclic carotenoid content that correlates with a reduction in *εLCY* mRNA levels.

### The *εLCY* promoter modulates chemical, developmental, and light-mediated regulation of reporter gene expression

**Fig. 2:**
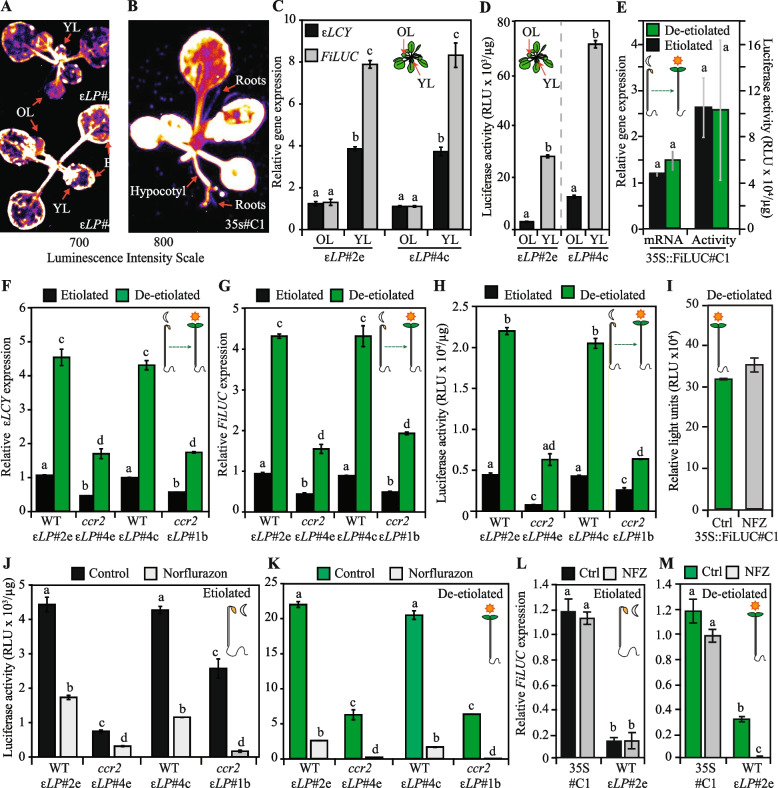
Quantification of *εLCY* promoter enabled *LUCIFERASE* expression and reporter activity in response to chemical, genetic, developmental, and environmental changes. **A**,**B** In planta luminescence emitted by independent WT transgenic plants harbouring the *εLCY*Prom-*εLCY*5′UTR::FiLUC (*εLP::FiLUC*#2e; *εLP*#2e, *εLP::FiLUC*#4c; *εLP*#4c) or CaMV35S::FiLUC (35S#C1) transgenes. The colour bar displays the intensity scale where black denotes no light emission and white indicates maximum luminescence. **C** *εLCY* and *FiLUC* gene expression levels in young leaves (YL) and old leaves (OL) from WT transgenic lines (*εLP*#2e and *εLP*#4c). **D** FiLUC activity (RLU/µg protein) in young and old leaves from WT transgenic lines (*εLP*#2e and *εLP*#4c). **E** Relative *FiLUC* mRNA expression and protein activity of WT etiolated and de-etiolated seedlings harboring the CaMV35S-reporter fusion (35S::FiLUC#C1). **F–H** *εLCY* (**E**) and *FiLUC* mRNA (**F**) and activity (**H**) levels in transgenic etiolated and de-etiolated transgenic WT (*εLP*#2e, *εLP*#4c) and *ccr2* (*εLP*#4e, *εLP*#1b) seedlings. **I** FiLUC activity in control (Ctrl) and 5 μM NFZ-treated 35S::FiLUC#C1 transgenic de-etiolated seedlings. **J**,**K** FiLUC activity in control and 5 μM NFZ-treated etiolated (**H**) and de-etiolated (**I**) transgenic WT (*εLP*#2e, *εLP*#4c) and *ccr2* (*εLP*#4e, *εLP*#1b) seedlings. **L**,**M** *FiLUC* mRNA expression in 5 μM NFZ-treated etiolated (**L**) and de-etiolated (**M**) transgenic WT seedlings harboring *CaMV35S* (35S#C1) and *eLCY* (*εLP*#2e) promoter-reporter fusions. Lettering denotes significance by a one- or two-way ANOVA statistical analysis with a post hoc Tukey test. Data is representative of two to three independent experiments, and standard error bars are shown (*n* = 3–9). RLU; Relative light units, *FiLUC*; Firefly intron-containing *LUCIFEREASE* gene

*εLCY* expression is regulated by *ccr2* and NFZ treatment, as well as during leaf development and seedling photomorphogenesis (Fig. 1J). To decipher potential feedback mechanism(s) regulating *εLCY* expression, an upstream fragment (− 450 bp; starting downstream of the 3′UTR from the adjacent *NMT1* gene; AT5G57020) of the *εLCY* promoter (plus the 5′UTR) was fused to the *FiLUC* reporter gene (*εLCY*Prom-*εLCY*5′UTR::*FiLUC; εLP*) and transformed into WT. Mature leaf tissues from transgenic lines (F_2_) harboring *εLP* enabled low luminescence (Av. 3511 relative light units; RLU) compared to the strong CaMV35S promoter (Av. 63,186 RLU) (Additional file 1: Figure S6A-B). The distribution in luminescence variation among independent *εLP* lines (23 ranged from 295 to 7661 RLU) was similar to CaMV35S (36 lines ranged from 480 to 220,692 RLU) due to transgene position effects [62]. In planta imaging of luminescence from two representative WT homozygous lines (WT-*εLP::FiLUC*#2e and WT-*εLP::FiLUC*#4c) revealed the *εLP* promoter was active in most tissues except the roots (Fig. 2A). The WT-*εLP::FiLUC* lines displayed lower luminescence in the fully expanded older leaves and stronger luminescence in the outer expanding regions from newly emerged leaves. In contrast, the *35S::FiLUC#C1* line enabled strong constitutive luminescence in leaves, cotyledons, petioles, hypocotyl, and roots (Fig. 2B). The transcript levels of *εLCY* and *FiLUC* in two *εLP::FiLUC* transgenic lines were four- and eight-fold more abundant in younger (leaves 1–5) relative to older (leaves 6–10) leaves from mature rosettes, respectively (Fig. 2C). FiLUC activity was, similarly, five- to seven-fold higher in younger compared to older leaves from both *εLP::FiLUC* transgenic lines (Fig. 2D). Therefore, the − 450-bp *εLCY* promoter fragment was responsive to changes in leaf development, enabling higher *FiLUC* levels in younger vs. older leaves.

The responsiveness of the *εLCY* promoter fragment to feedback signalling in *ccr2* etiolated and de-etiolated seedlings was investigated. The WT-*εLP::FiLUC*#2e and WT-*εLP::FiLUC*#4c transgene lines were introgressed with *ccr2.1* (*ccr2.1*-*εLP::FiLUC*#4e) and *ccr2.5* (*ccr2.5-εLP::FiLUC*#1B), respectively, to create homozygous isogenic lines. Etiolated and de-etiolated WT seedlings harboring the CaMV35S promoter-reporter (*35S*::*FiLUC#C1*) displayed similar *FiLUC* transcript and activity levels (Fig. 2E). Compared to WT, *FiLUC* (and *εLCY*) gene expression was significantly reduced (> 50%) in etiolated *ccr2* seedlings (Fig. 2F,G). Three days after constant illumination, de-etiolated WT-*εLP::FiLUC* (#2e and #4c) seedlings displayed a > four-fold increase in *FiLUC* as well as *εLCY* expression. The *εLCY* and *FiLUC* expressions were approximately 50% lower in *ccr2*-*εLP::FiLUC* etiolated and de-etiolated seedlings relative to their WT isogenic parental line (Fig. 2F,G). Fold changes in FiLUC activity enabled by the *εLCY* promoter in WT vs. *ccr2* and etiolated vs. de-etiolated seedlings mirrored the pattern of *FiLUC* (and *εLCY*) expressions (Fig. 2H). Therefore, gene expression enabled by the *εLCY* promoter was negatively regulated by feedback signalling in *ccr2* and activated by light during photomorphogenesis.

We next investigated the responsiveness of the *εLCY* promoter fragment to NFZ-induced retrograde signalling in etiolated and de-etiolated seedlings. NFZ did not alter FiLUC activity levels enabled by the CaMV35S promoter in de-etiolated transgenic seedlings (35S::FiLUC#C1; Fig. 2I). Luciferase activity in WT-*εLP::FiLUC* lines (#2e and #4c) was significantly repressed in de-etiolated (8.4- to 12.2-fold) and to a lesser extent etiolated (2.6- to 3.7-fold) seedlings treated with NFZ relative to controls (Fig. 2J,K). In *ccr2*-*εLP::FiLUC* lines (#4e and #1b), FiLUC activity was further suppressed by NFZ in etiolated (2.4- to 15.3-fold) and even more so in de-etiolated (27.3- to 99.6-fold) seedlings. NFZ did not impact *FiLUC* transcript levels enabled by the CaMV35S promoter in etiolated and de-etiolated transgenic seedlings (35S::FiLUC#C1; Fig. 2L,M). Intriguingly, NFZ did repress *FiLUC* expression in de-etiolated but not etiolated seedlings tissues from the WT-*εLP::FiLUC*#2e transgenic line (Fig. 2L,M). Therefore, the *εLCY* promoter was responsive to NFZ-induced retrograde signalling and suppressed *FiLUC* activity and expression in etiolated and de-etiolated seedlings, respectively indicating it can modulate both transcriptional and post-transcriptional processes in planta.

### The *εLCY* promoter harbours alternative transcription start sites and a conserved IRES motif within the most abundant 5′UTR fragment

**Fig. 3:**
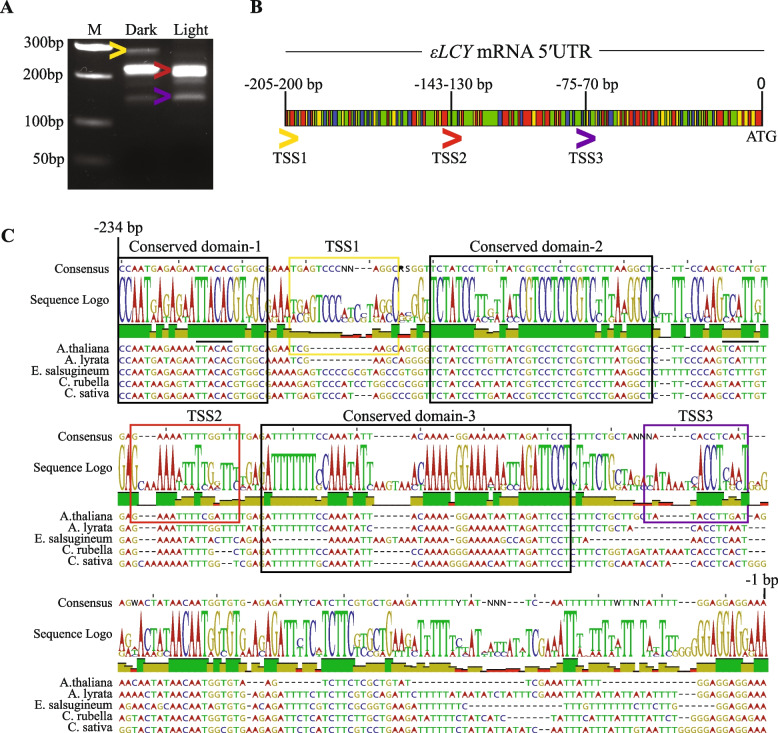
Analysis of *εLCY* mRNA transcription start sites and 5′UTR sequence homology among *Brassicaceae* species. **A** Gel electrophoresis showing Rapid amplification of cDNA ends (5′RACE) fragments that define the transcription start site (TSS) of the *εLCY* 5′UTR in etiolated (dark) and de-etiolated (light) grown wild-type seedlings. The electrophoresis image is representative of at least three independent 5'RACE experiments. **B** Sequence representation showing three alternative transcription start sites positioned around − 200, − 130, and − 70 bp upstream from the translational start codon sequence (ATG). The coloured arrowheads depicted below the sequence depict the starting positions of three alternative 5′UTR fragments, and the identifier bar depicts the nucleotide composition (Adenine; red, Thymine; green, Guanine; yellow, Cytosine; blue). **C** Sequence of the *εLCY* 5′UTR showing the alternative TSS positioned proximal to evolutionary conserved sequence domains (black box) identified among *Brassicaceae* species. Sequences of *Arabidopsis thaliana* (*A. thaliana*; NC_003076), *Arabidopsis lyrata* (*A. lyrata*; NW_003302548), *Eutrema salsugineum* (*E. salsugineum*; NW_006256829), *Capsella rubella* (*C. rubella*; NW_006238916), and *Camelina sativa* (*C. sativa*; NC_025702) were aligned using Geneious Prime Software (v10.2.6). NCBI identifiers of sequences analysed are shown above in brackets. The two black lines above the *A. thaliana* sequence (3–10 nucleotides upstream of TSS_1_ and TSS_2_ regions) denote homology to the consensus Initiator (Inr) element (Figure S7B-C)

The rapid amplification of cDNA ends (5′RACE) was used to determine the transcription start site(s) of the *εLCY* mRNA and facilitate the in silico analysis of the *εLCY* promoter fragment (− 450 bp) for regulatory motifs that could modulate feedback regulation in planta. Three distinct 5′RACE amplicon products and a minor amplified band immediately below the 200-bp fragment were detected in mRNA from etiolated (dark-grown) and de-etiolated (dark to light shift) seedling tissues (Fig. 3A, Additional file 1: Figure S7A). Sequencing these products revealed three clear alternative transcription start sites in dark-grown seedlings, each ranging within a small block of 5 to 13 bp nucleotides; TSS_1_ (− 200 to − 205 bp), TSS_2_ (− 130 to − 143 bp), and TSS_3_ (− 70 to − 75 bp) (Fig. 3B). TSS_1_ generated the longest 5′UTR fragment length of around − 200 bp, specific to dark-grown seedlings. TSS_2_ and TSS_3_ generated two 5′UTR fragments (− 133 and − 70 bp) in dark and light-grown seedlings, with TSS_3_ being less abundant. The − 133 bp 5′UTR fragment beginning at TSS_2_ was the most highly abundant 5′RACE amplicon, although a few varying shorter sequences were identified, keeping in agreement with the smaller amplicons smeared immediately below the 200-bp fragment (Fig. 3A). TSS_2_ aligned with the transcription start of three EST cDNA transcripts deposited into GenBank (BP866039, BX831396, and EH842927). Sequence alignment of − 234 bp of the *εLCY* 5′UTR from closely related *Brassicaceae* species (*A. thaliana, Arabidopsis lyrate*; *A. lyrata*, *Eutrema salsugineum*; *E. salsugineum*, *Capsella rubella*; *C. rubella*, and *Camelina sativa*; *C. sativa*) revealed similar sequence regions (referred to as a conserved domain; CD) in between and neighbouring the three *εLCY* transcription start sites (Fig. 3C). That is, CD-1 (− 234 to − 213 bp) was positioned upstream of TSS_1_, while CD-2 (− 190 to − 155 bp) was located downstream in between TSS_1_ and TSS_2_. CD-3 (− 124 to − 87 bp) was located downstream of TSS_2_ prior to TSS_3_ (Fig. 3B). Therefore, the − 450 bp *εLCY* promoter has three alternative transcription start sites and the − 133 bp 5′UTR fragment was most abundant in etiolated and de-etiolated seedlings.

In-silico analysis identified two distal TATA-like boxes (− 305 and − 357 bp) and CAAT boxes (− 234 and − 434 bp) considerably outside the consensus distance (− 20 to − 60 bp) defined for plant RNA Polymerase II promoters [63, 64] (Additional file 1: Figure S7B). Transcription of TATA-less promoters can operate with other core-promoter elements, such as the Initiator element (Inr), downstream promoter element (DPE), and the pyrimidine patch (YP), all of which align with the three *εLCY* transcription start sites (Additional file 1: Figure S7B-C). Inr sequence elements (Py-Py-A-N-T/A-Py-Py; where Py is C/T, and N is A,G,C,T) promote transcription initiation in plants by acting as recognition sites to bind TFIID [65, 66]. Two Inr motifs identified were positioned 3–10 nucleotides upstream from TSS_1_ (− 222 bp) and TSS_2_ (− 153 bp), aligning closely with the consensus range (− 14 to + 14 bp) defined for plant TATA-less promoters (Fig. 3C, Additional file 1: Figure S7B-C) [64, 67]. Two DPE elements (− 256 bp, − 343 bp) reported to function with Inr to bind TFIID were identified upstream from TSS_1_, yet are located outside the consensus distribution (+ 25 to + 35 bp) relative to the TSS of plant dicot promoters [63]. The YP is a core promoter element usually located upstream from the TSS (− 50 bp) and downstream from the TATA or INR motifs. Three YP motifs (− 179, − 86, and − 37 bp) were identified downstream of TSS_1_ (− 179 bp) TSS_2_ (− 86 bp), and TSS_3_ (− 37 bp) (Additional file 1: Figure S7B-C). Hence, the Arabidopsis *εLCY* promoter lacks a canonical TATA box and resembles a TATA-less promoter harboring interspaced core promoter initiator elements associated with alternative transcription start sites.

The distal portion of the *εLCY* promoter (− 450 to − 205 bp) did not harbour highly conserved *cis*-acting elements, and only the core region of abscisic acid (ABRE; − 444 bp) and light (G-box; − 213 bp, TCT-motif; − 279 bp) responsive motifs were identified. Overlapping TSS_2_ (− 137 bp) was an intron-mediated enhancer (IME)-like motif (TTNGATTTG; 88% homology) which is known to affect RNA polymerase processivity, transcription, nuclear export, transcript stability, and/or mRNA translation efficiency [68–70]. A highly conserved internal ribosome entry site (IRES) structural motif common to RNA viruses was identified using UTRscan to span the TSS_2_ 5′UTR (− 1 to − 82 bp) starting downstream of CD-3. A putative upstream open reading frame (uORF) (Met-Val-STOP; − 47 to − 39 bp) was found within the IRES motif (Additional file 1: Figure S7C). IRES elements can adopt structural configurations and function to recruit ribosomes, initiation factors, RNA-binding proteins (RBPs), and/or capture ligands associated with maintaining metabolic homeostasis [45, 71, 72]. Therefore, while we cannot exclude a regulatory motif(s) within the TATA-less initiator zone upstream from TSS_1_, the − 133 bp 5′UTR harbouring a conserved IRES and uORF has potential to modulate feedback regulation of the *εLCY* promoter in planta.

### Disruption of the uORF within the IRES enhances CaMV35S enabled FiLUC activity

**Fig. 4:**
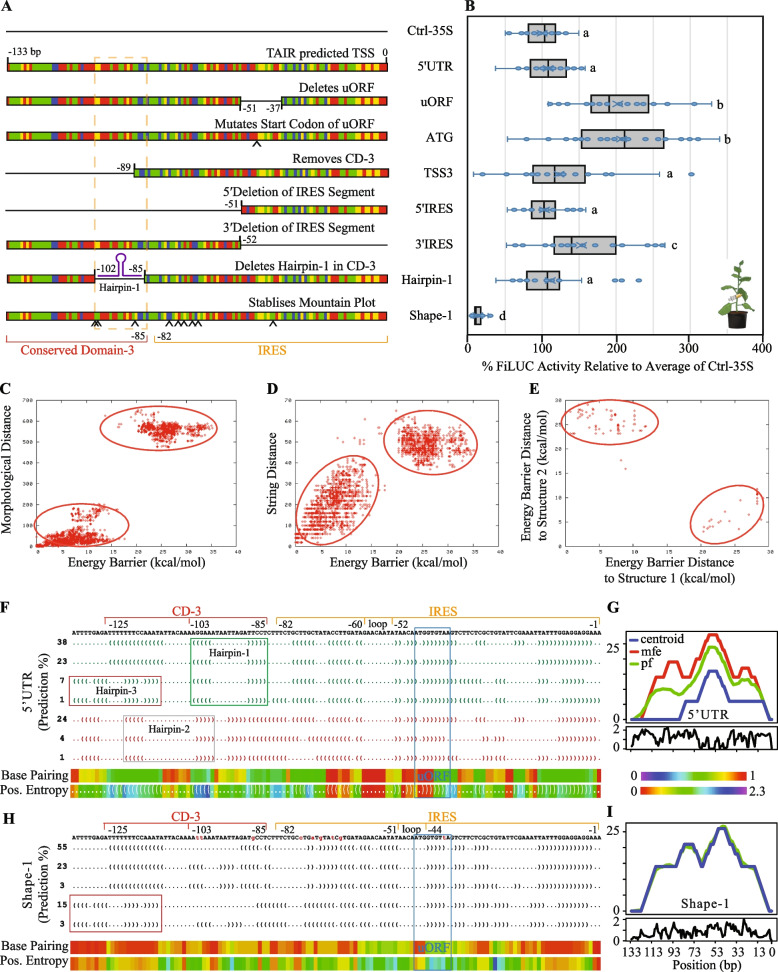
RNA structural analysis of the *εLCY* 5′UTR and regulation of promoter-reporter gene activity in transgenic *Arabidopsis* and transiently-infected tobacco leaves. **A** Diagrammatic representation of the various synthetic mutations and deletions (MutDels) of the *εLCY* 5′UTR fused in between the CaMV35S promoter and intron-containing *FIREFLY LUCIFERASE* (*FiLUC*) reporter gene. Constructs include the Ctrl-35S (Control CaMV35S promoter without 5′UTR), 5′UTR (TSS predicted by 5’RACE and TAIR10 prediction; − 133 to − 1 bp upstream from *εLCY* mRNA start codon), and MutDels; uORF (deletes upstream open reading frame; − 51 to − 37 bp), ATG (mutates start codon of uORF from ATG to AAG), TSS3 (removes conserved-domain 3; CD-3, − 90 to − 1 bp, and retains entire TSS_3_ fragment), 5′IRES (Removes 5′ segment of IRES and CD-3; − 133 to − 51 bp), 3′IRES (Removes 3′ segment of IRES; − 52 to − 1 bp), Hairpin-1 (deletes stem-loop structure in CD-3; − 102 to − 85 bp), and Shape-1 (9 serial substitution mutations predicted to stabilise a single RNA structural prediction). Colour bars represent nucleotides (A; red, T; green, C; blue, G; yellow) and ^ denotes mutations. **B** FiLUC activity in agrobacteria-infiltrated tobacco leaf tissues transiently expressing promoter-reporter gene constructs. An in vivo leaf disc bioassay [93] was used to quantify luciferase activity in leaves from 8 to 12 plants and three independent experiments. The Relative Light Units (RLU) emitted per leaf per construct was calculated as a percentage relative to the average of Ctrl-35S within an experiment. The box plot shows the median (central line marks the mid-point) and outer box lines as well as error bars mark the four quartile data marks. Data points represent individual leaves (*n* = 21–31). Lettering denotes a statistical one-way analysis of variance followed by a post hoc Tukey’s test (*p* < 0.05). **C–E** Structural conformational prediction of the *εLCY* 5′UTR (− 133 bp) using paRNAss software to show the morphological distance plot (**C**), string distance plot (**D**), and energy barrier distance to structures (**E**). The alignment distance measures the predicted structures’ structural similarity to convert between different RNA structures. The cluster of each kinetically related structure with the same consensus folding conformation forms a separate energy cloud. There must be at least two energy clouds with a distinct separation on the *x*- and *y*-axis of the distance plot to indicate a potential to switch between distinct structural predictions. **F**, **H** RNAShapes analysis of the 5′UTR (**F**) and shape-1 (**H**) sequences showing Vienna shape representatives (dot-bracket notation; “(” and “.” represent paired and unpaired bases respectively), probability prediction (%), base-pairing probability colour chart (1 implies highly stable), and positional entropy colour chart (0 indicates base-pair is always unpaired or paired with its partner) of the most probable structures generated by RNAfold software default settings. Green or red dot-bracket notations denote similar representations. Red and orange lines above the *εLCY* 5′UTR sequence denote Conserved Domain-3 (CD-3) and IRES. Boxed regions denote hairpin structures. **G**, **I** RNAfold mountain plot representations showing the thermodynamic ensemble of RNA structures of the *εLCY* 5′UTR (**G**) and shape-1 (**I**) sequences. The mountain plot depicts the number of base pairs enclosing a sequence position (average for base-pair probabilities) versus the sequence position to help visualize the MFE structure, centroid structure, and base-pairing probabilities of the predicted RNA structure and its reliability. “mfe” represents the minimum free energy structure; “pf” indicates partition function; “centroid” represents the best average structure in a plot of height m(k) versus position (bp), where the height is given by the number of base pairs enclosing the base at a position. Loops correspond to plateaus (hairpin loops are peaks), and helices to slopes. Entropy of each bp along the RNA sequence (− 1 to − 133 bp) is shown below the mountain plot and lower entropy values indicate higher stability of RNA secondary structures

A gain-of-function approach was used to interrogate the *εLCY* 5′UTR fragment (starting at − 133 bp, TSS_2_ identified by 5′RACE) by testing mutation and deletion (MutDel) versions placed in between the CaMV35S promoter and the *FiLUC* reporter gene. The CaMV35S promoter served as the control (Ctrl-35S) and MutDels of the *εLCY* 5′UTR tested potential functions for the (1) upstream open reading frame (-uORF; removed the entire 3 amino acid peptide, -ATG; disrupts the start codon sequence), (2) CD-3 and alternative transcription (-TSS3), and (3) IRES (−5′IRES and −3′IRES) (Fig. 4A, Additional file 1: Figure S7D). Luciferase activity was quantified in agro-infiltrated tobacco leaves transiently expressing promoter-reporter fusions (35S::FiLUC, 35S-5′UTR::FiLUC, 35S-MutDels::FiLUC) fusions and relative light units were expressed as a percentage relative to Ctrl-35S that had no 5′UTR (Fig. 4B)*.* The − 133 bp unmodified (native) 5′UTR fragment did not affect FiLUC activity enabled by the CaMV35S promoter. Removal of the three amino acid peptide (-uORF) and mutation of the initiation start codon (-ATG) caused a significant two-fold increase in FiLUC activity. Removal of CD-3 and hence the TSS_3_ fragment (-TSS_3_) did not impact luciferase activity relative to the − 133 bp *εLCY* 5′UTR. Deletion of the 3′ (−3′IRES; also lacks the uORF) but not 5′ (−5′IRES; contains uORF) end of the IRES caused a subtle increase in FiLUC activity (Fig. 4B). Overall, while the native *εLCY* 5′UTR did not affect CaMV35S promoter regulation, disruption of the open reading frame (uORF, ATG, 3′IRES) upregulated FiLUC activity revealing a more intricate mechanism linked to the IRES.

### The 5'UTR exhibits RNA structures that are stable within IRES and alternate at CD-3

IRES-mediated regulation is associated with a conformational change in RNA secondary structure definition. To investigate the possibility of RNA secondary structural rearrangements, we bioinformatically analysed the most abundant − 133 bp (TSS_2_) *εLCY* 5′UTR fragment using a suite of RNA structural prediction programs. paRNAss software was used to interrogate *εLCY* 5′UTR alternate RNA secondary structure folding spaces within an energy range above the minimal free energy (MFE) [20, 73, 74]. Two distinct and separate energy clouds were displayed on plots of morphological distance, string distance, and validation of energy barrier clustering indicating energetically separable RNA structural populations—one near the *x*-axis and another near the *y*-axis (Fig. 4C–E).

To ascertain the potential to switch between structural configurations a command-line version of Infernal (1.1rc1, cmscan, default parameters) was used to scan *εLCY* 5′UTR (AT5G57030; Chr5:23,077,255–23080053) sequence against the Rfam database collection for RNA families. The search stringency was reduced resulting in identifing weak homology (E-value *p* < 0.07) between an SMK_box riboswitch (RF01767; 81 bp) and the *εLCY* 5′UTR (− 96 to + 13 bp of mRNA) fragment overlapping the IRES motif (Additional file 3; Dataset S1). We undertook a modelled approach to ascertain if the *εLCY* 5′UTR had other RNA structural characteristics shared with 27 different classes of metabolite-binding RNA riboswitches found in bacteria (Additional file 4: Dataset S2). Bacterial nucleic acid sequences retrieved from the Rfam database were analysed using RNAshapes software to quantify the number of shapes (between 1 to 5; *p* > 0.1 or 10%), variants per shape, minimum/maximum of shape forming probabilities (%), and minimal free energy (mfe; kcal) for 2691 RNA riboswitches (Table 1). Most riboswitches displayed one to two structural shape definitions (35 and 42%, respectively), and a considerable proportion (23%) displayed between three to five structures. RNAShapes software [75] revealed seven distinct RNA structural predictions (> 1% probability) within the *εLCY* 5′UTR (− 133 bp) having a minimal free energy (mfe) ranging from − 19.1 to − 22.4 kcal/mol. There were three prominent structural definitions (38, 23, and 24% probability) identified between the minimum (11%) and maximum (58%) probabilities displayed by 457 variants from 25 riboswitch classes (Fig. 4F; Table 1). There were two major RNA structural definitions, one dominating 38% and 23% (green font) and an alternative 24% (red font) of probable structural classes. All structural predictions had a central loop (− 52 to − 60 bp) within the IRES motif (− 1 to − 82 bp) and were distinguished by hairpin configurations within CD-3 (Fig. 4F, Additional file 1: Figure S8A). Therefore, bioinformatically, the 5′UTR fragment exhibits two highly probable RNA structural definitions that alternate within CD-3.

**Table 1:** RNAshapes modelling of structural features from 27 different classes of bacterial metabolite-binding RNA riboswitches

Number of shapes (*p* > 0.1)	Number of variants	Shape potential (%)	Shape-forming probability (%)		Minimal-free energy (kcal)	
			Minimum	Maximum	Minimum	Maximum
1	948	35	77	100	− 81	− 6
2	1123	42	20	78	− 79	− 6
3	457	17	11	58	− 77	− 6
4	143	5	11	45	− 82	− 14
5	20	1	10	29	− 68	− 24

The RNAfold [76, 77] mountain plot representation in a plot of height versus position confirms a prominent centroid structural peak (− 58 bp) within the IRES loop surrounded by a higher probability of base-pairing at the uORF and lower minimal positional entropy indicative of fewer secondary structure outcomes within this probability space (Fig. 4G). In contrast, the CD-3 region (− 85 to − 125) lacked a centroid structure, the mfe and partition function (pf) structural predictions were displayed as separate peaks, and there was a reduced probability of base-pairing with higher positional entropy surrounding the CD-3 hairpin configurations (Fig. 4F,G). Hairpin-1 (− 85 to − 103 bp) and hairpin-2 (− 98 to − 120 bp) were distinguished in four dominant and three alternative structural classes, respectively representing 69% and 29% of all probabilities. Whereas hairpin-3 (− 97 to − 133 bp) was identified in only two of the four dominant structural probability configurations representing 8% of structural predictions (Fig. 4F, Additional file 1: Figure S8A). Taken together, the IRES motif harboring an uORF exhibits a stabile RNA structural definition, while CD-3 could potentially switch between alternative RNA hairpin shape configurations.

### Stabilisation of a single 5′UTR RNA structural definition represses FiLUC activity

We tested whether the alternative 5′UTR RNA structural definitions within CD-3 adjacent to the IRES can modulate promoter-reporter activity. The hairpin-1 (− 85 to − 103 bp) feature was removed from CD-3 within the 5′UTR, and transient infection of tobacco leaves with this MutDel fragment inserted between the 35S promoter and FiLUC reporter did not affect luciferase activity relative to unmodified *εLCY* 5′UTR (Fig. 4A,B, Additional file 1: Figure S7D). Nine serial mutations were introduced into the 5′UTR sequence (referred to as shape-1) to stabilise the mountain plot representation with negligible separation between the structure prediction curves (centroid, partition function; pf, and mfe) (Fig. 4I, Additional file 1: Figure S7D). Shape-1 displayed three similar structural probabilities of 55, 23, and 3%, representing 81% of all predicted structural definitions. Shape-1 structural predictions retained similar dot-bracket notations surrounding the IRES central loop (− 44 to − 51 bp) that was slightly offset position compared to the native 5′UTR (compare Fig. 4F, H). Within the shape-1 CD-3 region, hairpin-1 and hairpin-2 were removed, hairpin-3 was stabilised in 18% of structural probabilities, exhibiting a higher base pairing probability of binding with lower positional entropy compared to the 5′UTR (Fig. 4F–I, Additional file 1: Figure S8B).

The structurally stable shape-1 fragment repressed 35S enabled FiLUC activity by 8.2-fold relative to the unmodified 5′UTR in the transient *N. tabacum* (tobacco) leaf disc bioassay (Fig. 4A,B). The shape-1 (35S-shape1::FiLUC), 5′UTR (35S-5′UTR::FiLUC), and 35S (35S::FiLUC) binary vectors were transformed into WT and *ccr2* germplasm. Independent homozygous transgenic lines (T_3_) harboring 35S, 5′UTR, and shape-1 promoter-reporter transgenic lines displayed a normal distribution in foliar luminescence variability similar to that of the 35S promoter, likely due to a transgene position effect (Additional file 1: Figure S6B-C). The average luciferase activity of all lines harboring the 35S promoter with and without the 5′UTR was similar, and 7.2- to 7.9-fold higher than shape-1, respectively (Additional file 1: Figure S6C). Therefore, nine serial mutations predicted to stabilize 5′UTR RNA structural definitions within CD-3 adjacent to the IRES have imparted a negative expression platform affecting CaMV35S promoter activity.

### Stabilisation of the 5'UTR hairpin-3 definition promotes post-transcriptional regulation

Mutations within shape-1 were sequentially restored back to their native WT sequence to interrogate if RNA structural changes within CD-3/IRES regions, and/or the gain/disruption of a novel *cis*-acting element had imparted the negative expression platform (Additional file 1: Figure S7D). Shape-2 (8 mutations), shape-6 (3 mutations), and shape-7 (2 mutations; − 102 bp, GG > TT) displayed negligible separation between the structure prediction curves in their mountain plot representations (Additional file 1: Figure S9A). The dot-bracket notations indicated that the central IRES loop region in shape-6 and shape-7 was surrounded by a high probability of base pairing and lower entropy that resembled the native 5′UTR (Additional file 1: Figure S9B-D). Shapes-2/6/7 were not predicted to form hairpin-1 or hairpin-2 and had the highest probability of forming hairpin-3 (− 97 to − 133 bp) within CD-3 (Additional file 1: Figure S8, S9B). Hairpin-3 dominated 85% of the structural definitions of shape-7 that harboured only two mutations (GG > TT) disrupting hairpin-1 and hairpin-2 formation (Additional file 1: Figure S7D, S8E, S9B-D).

**Fig. 5:**
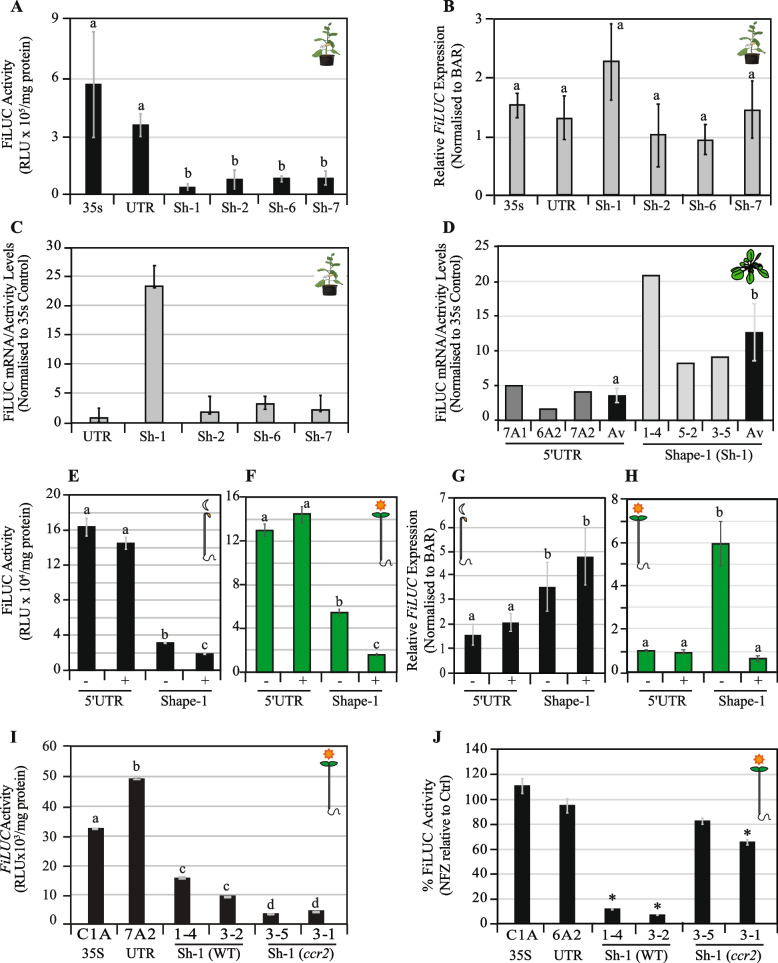
LUCIFERASE activity and mRNA levels in leaves and seedlings enabled by *eLCY* 5′UTR and shape regulation of the 35S promoter. **A–C** Quantification of transient FiLUC activity (RLU/mg protein) (**A**), relative *FiLUC* expression (**B**), and *FiLUC* mRNA to protein activity ratios (**C**), in agro-infiltrated tobacco leaves harboring 35S, 5′UTR, and shape structural definition variants (shape-1, −2, −6, −7). The *BAR* (Basta-resistance) gene was used to normalise between foliar agro-infiltrations, and the *ACTIN* housekeeping gene was used to normalise for biological variation. Promoter constructs tested include 35S::FiLUC (*35S*), 35S-5′UTR::FiLUC (UTR), 35S-shape1::FiLUC (Sh-1), 35S-shape2::FiLUC (Sh-2), 35S-shape6::FiLUC (Sh-6), 35S-shape7::FiLUC (Sh-7). Data represents the average of two independent experiments and six biological replicates with standard error bars shown (*n* = 3–6). **D** *FiLUC* mRNA to protein activity ratios in leaves from independent *Arabidopsis* transgenic seedling lines (T_3_) harboring 5′UTR or shape-1 fragments fused between the 35S promoter and FiLUC reporter fusions treated with (+) and without (−) norflurazon. Relative *FiLUC* expression (normalised to *PP2A*) and FiLUC activity (RLU/mg protein) were quantified in rosette leaves from three plants/transgenic line. The control CaMV35S (35S::FiLUC#C1) line was used to normalise mRNA and activity levels among individual 5′UTR (35S-5′UTR::FiLUC; #7A1, #6A2, #7A2) and shape-1 (35S-shape1::FiLUC; #1–4, #5–2, #3–5) transgenic lines. The average and standard error of ratios from three independent lines are displayed (*n* = 3). **E–H** FiLUC activity (RLU/mg protein) (**E**,** F**) and relative *FiLUC* mRNA expression (normalised to *PP2A*) (**G**.**H**) in etiolated (**E**,** G**) and de-etiolated (**F**,** H**) transgenic *Arabidopsis* seedlings harbouring the 5′UTR (35S-5′UTR::FiLUC#7A1) and shape-1 (35S-shape1::FiLUC#1–4) promoter-reporter fusions. Data is representative of multiple experiments (*n* = 3). **I**,**J** FiLUC activity (RLU/mg protein) in 35S, 5′UTR, and/or Shape-1 (Sh-1) WT and *ccr2* transgenic seedlings (**I**) and percentage (%) change in FiLUC activity following norflurazon-treatment of de-etiolated seedlings (**J**). Mean and standard error (*n* = 3) are displayed, data is representative of two independent experiments. Lettering denotes statistical significance (one-way ANOVA, *p* < 0.05)

The 35S and 5′UTR, shape-1 (9 mutations), shape-2 (8 mutations), shape-6 (3 mutations), and shape-7 (2 mutations) promoter-reporter fusions were transiently expressed in tobacco leaf tissues, and FiLUC activity and mRNA levels were quantified. The shape fragments (shape-1, −2, −6, −7) showed a significant 4.6 to 12.5-fold reduction in luciferase activity compared to the unmodified 5′UTR fragment, which was not significantly different from the CaMV35S promoter without any 5′UTR (Fig. 5A). Intriguingly, the *FiLUC* transcript levels were similar in tobacco leaves transiently expressing promoter-reporter fusions harboring the modified shape fragments (shape-1, −2, −6, −7), unmodified 5′UTR and without any 5′UTR (35S) (Fig. 5B). Therefore, all the shape variants (shape-1, −2, −6, −7) confer a negative regulating post-transcriptional expression platform that associates with the stabilisation of a hairpin-3 RNA structural definition within CD-3 upstream of the prominent centroid peak within the IRES.

The regulatory mechanism was further investigated by comparing the *FiLUC* mRNA to FiLUC activity ratio (mRNA/activity) for native 5′UTR and modified shape fragments relative to 35S using tobacco transient (tobacco) and transgenic (Arabidopsis) leaf promoter-reporter expression systems. In tobacco, the native 5′UTR mRNA/activity ratio did not significantly change, indicating it does not affect 35S promoter function. The ratios were slightly enhanced by shape-2 (1.8-fold), shape-6 (3.1-fold), and shape-7 (2.2-fold) in tobacco due to lower protein activity levels. Strikingly, the shape-1 ratio was exuberated by 23.2-fold in tobacco (Fig. 5C). Next, the shape-1 ratio was further investigated using multiple independent Arabidopsis transgenic plants harboring 35S, native 5′UTR, and modified shape-1 promoter-reporter fusions. The average foliar luminescence enabled by four 35S lines (Av. 194,744 RLU) was similar to eleven 5′UTR lines (Av. 211,387 RLU) and significantly higher than fifteen independent shape-1 lines (Av. 26,648 RLU) (Additional file 1: Figure S6C). Three unmodified 5′UTR lines displayed a higher foliar FiLUC mRNA/activity ratio ranging from 1.6 to 5.0-fold (Av. 3.5-fold), while three independent shape-1 lines displayed a 12.7-fold higher mRNA/activity ratio (Fig. 5D). One of the shape-1 transgenic Arabidopsis lines (#1–4) displayed a 20.8-fold higher FiLUC mRNA/activity ratio due to higher *FiLUC* transcript and lower FiLUC activity levels (Fig. 5C,D). Therefore, shape-1 post-transcriptionally repressed FiLUC activity and slightly enhanced transcript levels resulting in a higher FiLUC mRNA/activity ratio in both tobacco (transient) and Arabidopsis (transgenic) leaves.

### Shape-1 modulates transcriptional and post-transcriptional responses to feedback signalling

The shape-1 regulation was further investigated by evaluating the effect of NFZ-induced retrograde feedback signalling in transgenic Arabidopsis etiolated and de-etiolated seedlings. The native 5′UTR lines were unresponsive to NFZ treatment (+), displaying similar FiLUC activity and transcript levels to their untreated controls without NFZ (−) (Fig. 5E–H). In the absence of NFZ, the shape-1 FiLUC activity levels were significantly lower in etiolated (5.2-fold) and de-etiolated (2.4-fold) seedlings relative to the 5′UTR (Fig. 5E,F). Interestingly, the shape-1 *FiLUC* transcript levels were significantly higher in control etiolated (2.3-fold) and de-etiolated (5.8-fold) seedlings compared to the untreated and native 5′UTR (Fig. 5G,H). This supports findings that shape-1 drives a higher mRNA/activity ratio (Fig. 5C,D). NFZ treatment significantly repressed shape1 FiLUC activity levels in both etiolated (1.7-fold) and de-etiolated (3.5-fold) seedlings (Fig. 5E,F). In contrast, NFZ did not change shape-1 *FiLUC* transcript levels in etiolated seedlings but substantially repressed *FiLUC* transcript levels in de-etiolated seedlings by nine-fold relative to untreated shape-1 seedlings (Fig. 5G,H). Taken together, the peculiar shape-1 fragment imparted new functions to the 35S promoter activity, enabling (1) higher *FiLUC* transcript levels, (2) lower FiLUC protein activity levels, and (3) responsiveness to NFZ-induced feedback signalling in dark and light-grown seedlings.

The post-transcriptional feedback regulation modulated by the shape-1 was further investigated in independent WT and *ccr2* transgenic lines harbouring the 35S-shape1::FiLUC promoter-reporter fusion. The foliar luminescence levels in WT and *ccr2* genotypes habouring shape-1 were > five-fold lower on average than the native 5′UTR and CaMV35S promoters (Additional file 1: Figure S6C). Shape-1 WT (#1–4, #3–2) and *ccr2* (#3–5, #3–1) lines showed similar luminescence in mature leaves from plants grown under a long 16-h photoperiod which allows efficient photoisomerization of prolycopene to lycopene unlike in de-etiolated seedlings (Additional file 1: Figure S1, S6C) that are prone to *ccr2*-mediated feedback signalling (Fig. 2). FiLUC activity enabled by 35S-shape-1 lines was significantly lower in *ccr2* compared to WT and regardless of the genotype (WT vs *ccr2*), shape-1 displayed considerably lower FiLUC activity compared to the native 5′UTR (Fig. 5I). While NFZ treatment did not affect FiLUC activity enabled by the 35S with or without the native 5′UTR, it significantly suppressed FiLUC activity by > 89% in WT shape-1 lines, while only causing a subtle decline of 18 to 35% in *ccr2* shape-1 lines (Fig. 5J). Therefore, unlike the native 5′UTR the peculiar shape-1 fragment rendered the 35S promoter responsive to NFZ and *ccr2*-mediated feedback signalling in de-etiolated seedlings.

## Discussion

### The regulation of *εLCY* expression does not correlate with *cis*-carotene mediated signalling

A *cis*-carotene-derived ACS was shown to regulated plastid biogenesis, *HY5*, *PIF3*, and *PhANG* expression in *ccr2* etiolated and de-etiolated seedlings, as well as young emerging leaves [10, 12, 61]. We questioned whether feedback regulation of *εLCY* expression was linked to linear *cis*-carotene accumulation. Etiolated seedling tissues from *ccr2*, *ziso*, *det1-1*, and WT treated with NFZ, accumulated linear *cis*-carotenes and displayed reduced *εLCY* expression. The loss-of-function in ZISO (*ziso-155*) and impaired DET1 activity (*det1-154*) were previously shown to reduce linear *cis*-carotene levels in *ccr2* etiolated seedlings and restore PLB formation and *PhANG* expression by limiting the production of a yet-to-be-identified *cis*-ACS [10]. When compared to WT, the *εLCY* expression remained suppressed in *ccr2 ziso* and *ccr2 det1-154* etiolated seedlings despite a reduction in di-*cis*-ζ-carotene, neurosporene, and tetra-*cis*-lycopene, which are proposed substrates of the *cis*-ACS. We cannot rule out phytoene or phytofluene as substrates of another *cis*-ACS signal, since they accumulate in *ccr2*, *ziso*, *det1-1*, *ccr2 ziso*, and *ccr2 det1-154* germplasm as well as NFZ-treated WT tissues. However, the hyperaccumulation and decline of phytoene and phytofluene, respectively, in NFZ-treated *ccr2* did not further impact *εLCY* expression. Therefore, the accumulation of linear *cis*-carotenes and the yet-to-be-discovered *cis*-ACS do not appear to trigger feedback regulation of *εLCY* expression during skotomorphogenesis.

### Changes in cyclic carotenoids correlate with feedback regulation of *εLCY* expression

**Fig. 6:**
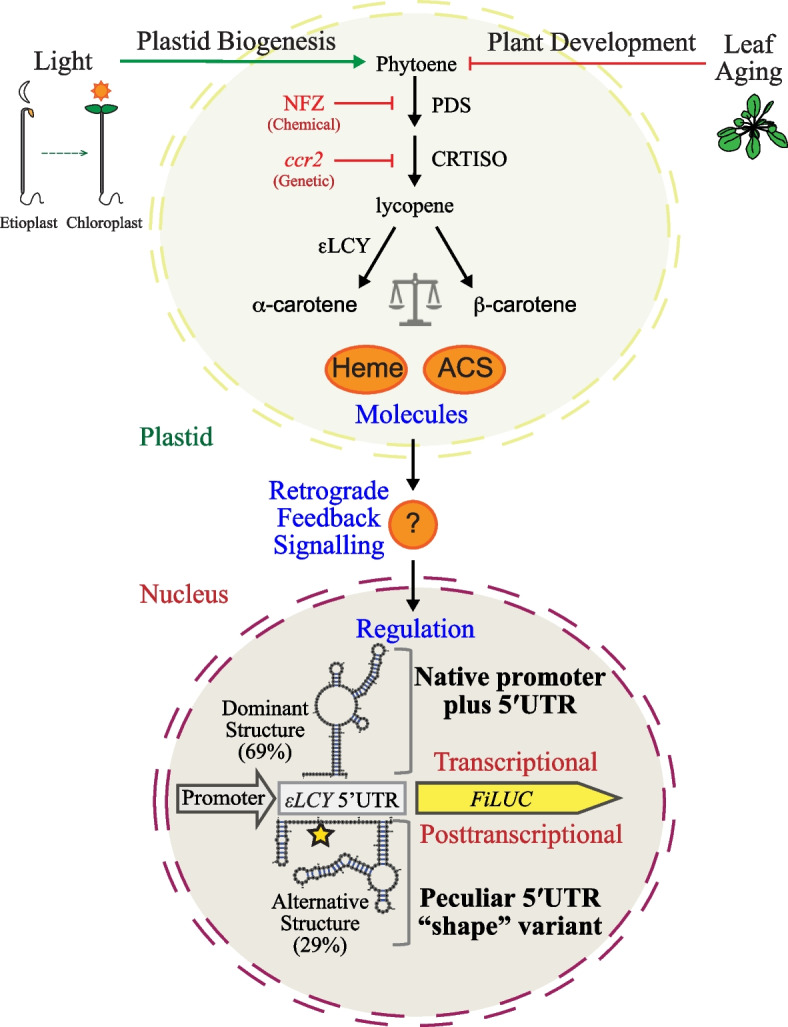
Model showing feedback signalling from the plastid and regulation of the *εLCY* promoter including the 5′UTR within the nucleus. *εLCY* modulates flux through the α/β-carotene branch in the pathway to balance carotenoid homeostasis during plastid biogenesis. *εLCY* expression levels within the nucleus correlate with changes in β-branch carotenoid levels in plastids from dark (etioplast) and light (chloroplast) grown *Arabidopsis* seedlings. Cyclic carotenoids and *εLCY* expression increase during seedling de-etiolation (light stimulated photomorphogenesis) and decrease during rosette leaf aging (development). The loss-of-function in CRTISO activity (*ccr2*) suppressed β-branch carotenoid biosynthesis and *εLCY* expression in etiolated and de-etiolated seedlings. Norflurazon (NFZ) inhibits PHYTOENE DESATURASE (PDS) activity, cyclic carotenoid biosynthesis, and suppresses *εLCY* expression. NFZ and *ccr2* trigger the generation of retrograde molecules in the plastid (e.g., heme and/or apocarotenoid signal; ACS) that signal control over events in the nucleus (either directly or indirectly via a phytohormone, miRNA, or protein; represented as a questions mark). *εLCY* and *LUCIFERASE* (*FiLUC*) expressions enabled by the native *εLCY* promoter plus 5′UTR were responsive to chemical (NFZ), genetic (*ccr2*), environmental (de-etiolated light vs. etiolated dark grown seedlings), and/or developmental (leaf aging, recently emerged young vs. mature expanded older leaves) cues. The *εLCY* 5′UTR harbours hallmark RNA structural features. The dominant native structural (69%) and alternative peculiar shape variant (29%) forms above and below the *εLCY* 5′UTR, respectively associate with transcriptional and/or post-transcriptional mechanisms, which are not exclusive and can cooperate to fine-tune gene expression. Mutations (yellow star) stabilised the *εLCY* 5′UTR probability of forming the alternative hairpin-3 structural configuration referred to as shape-1, that enabled responsive of the CaMV35S promoter to retrograde feedback signalling triggered by *ccr2* and NFZ-treatment at both the transcriptional and post-transcriptional levels of regulation, in a light-dependent manner

Genetic manipulations, dark to light shifts, chemical treatments, and developmental change in *Arabidopsis* tissues showed a correlation between changes in β-branch carotenoids and *εLCY* transcript levels (Additional file 2: Table S1). NFZ treatment of seedlings blocks downstream carotenoid biosynthesis and elicits a retrograde signal that suppresses *PhANG* expression to thereby impair plastid biogenesis [78]. *ccr2* also blocks carotenoid accumulation in etiolated tissues, yet the lack of any PLB formation in *ccr2* etioplasts and a unique transcriptomic profile indicated that *ccr2* could trigger both independent and overlapping signalling pathways [10, 61]. While *εLCY* was not considered a traditional *PhANG* like *LHCB1*, our evidence reveals that both these genes are repressed in NFZ-treated etiolated seedlings. Unlike NFZ-treated tissues, chloroplast biogenesis and carotenoid biosynthesis are delayed in *ccr2* de-etiolated seedlings as well as in young emerging leaf tissues, both of which exhibit lower *εLCY* expression levels indicative of feedback regulation. The suppression of *εLCY* expression by RNAi or mutations redirects metabolic flux towards the β-branch of the carotenoid biosynthesis pathway [32, 33, 37, 79]. Similarly, the exogenous expression of the *Pantoea ananatis* phytoene desaturase (*PaCRTI*) gene in wild-type, *tangerine*, or *old gold crimson* tomatoes increased β-carotenoid levels as well as *εLCY* expression during fruit ripening [25]. Here, we demonstrated exogenous expression of *PaCrtI* or the loss-of-function in CCD4 in *ccr2* etiolated tissues increased β-branch carotenoids (β-carotene and zeaxanthin) and induced higher *εLCY* transcript levels. Overall, the regulation of *εLCY* expression correlates well with changes in β-branch carotenoid levels that occur in response to chemical (NFZ), genetic (*ccr2*), developmental (aging leaves), and environmental (dark to light) changes (Additional file 1: Figure S1). It is possible that a plastid-derived retrograde signal (e.g., Heme), hormone, protein, or mRNA could feedback to regulate *εLCY* expression [16, 78]. Our favoured hypothesis is that a β-carotenoid-derived apocarotenoid signal (ACS) represents a likely candidate to regulate *εLCY* and balance flux through the alpha- and beta-branches in the carotenoid pathway during plastid biogenesis (Fig. 6).

### The *εLCY* promoter modulates transcriptional and post-transcriptional feedback regulation

We investigated the mechanism by which feedback signalling regulates *εLCY* expression. Retrograde signalling molecules can alter transcription and/or post-transcriptional processes to moderate feedback regulation in various plastid types [9, 78, 80]. Our analysis of two-independent isogenic WT and *ccr2* transgenic lines harbouring a − 450 bp *εLCY* promoter fragment (including 5′UTR) fused to *FiLUC* revealed that reporter activity levels mirrored *εLCY* expression during genetic (WT vs *ccr2*), environmental (photomorphogenesis), and chemically (NFZ treatment) triggered feedback regulation. That is, transcription enabled by the *εLCY* promoter like *εLCY* expression was suppressed by ~ 50% in *ccr2* compared to WT during skotomorphogenesis and substantially enhanced by ~ 4.5-fold during photomorphogenesis. While WT and *ccr2* seedlings display similar morphological and growth characteristics during etiolation and de-etiolation, *ccr2* generates a yet-to-be-defined *cis*-carotene-derived ACS that presumably can feedback to signal changes in nuclear gene expression [10, 12, 61]. Similarly, NFZ blocks β-branch carotenoid biosynthesis in both dark- and light-grown WT seedlings, triggering a retrograde signal that suppresses *PhANG* expression and impairs plastid biosynthesis [16]. NFZ suppressed *εLCY* and *εLP*::*FiLUC* mRNA levels in de-etiolated seedlings during photomorphogenesis*.* Seedling de-etiolation and NFZ treatment did not alter 35S::*FiLUC* expression or activity. Hence, the − 450 bp *εLCY* promoter, including 5′UTR, enabled responsive to NFZ-induced and *ccr2*-mediated feedback signalling predominantly at the transcriptional level during photomorphogenesis (de-etiolated seedlings). However, during skotomorphogenesis (seedling etiolation), the *εLCY* promoter, including the 5′UTR, conferred transcriptional and post-transcriptional modulation of *FiLUC* in response to *ccr2*-mediated and NFZ-induced feedback signalling, respectively. While the majority of *εLCY* promoter regulation occurred at the transcriptional level, possibly mediated by transcription factors, the post-transcriptional regulation induced by NFZ treatment in etiolated seedlings reveals a more complex mechanism that underpins feedback regulation of εLCY to rate-limit biosynthesis between the alpha- and beta-branch in carotenoid biosynthesis.

### The *εLCY* promoter includes a prominent 5'UTR haboring a conserved IRES motif and putative uORF

The *εLCY* −450 bp promoter lacked consensus transcription factor motifs known to modulate phytohormone responses associated with carotenoid feedback regulation [4, 5]. However, the first half of the *εLCY* promoter contained three distinct transcription start sites separated by domains conserved in *Brassicaceae* species, indicating functional variants of the 5′UTR can be produced in a light-dependent manner during seedling morphogenesis. The longest TSS_1_ (− 205 to − 200 bp) and shortest TSS_3_ (− 75 to − 70 bp) 5′UTR fragments were barely detected in etiolated seedlings. TSS_2_ (− 143 to − 130 bp) had a proximal Inr motif (− 152 to − 147 bp), pyrimidine/YP patches (− 179 and − 86), and an overlapping IME-like element (− 130 to 137 bp) able to recruit RNA polymerase II and enrich this most abundant 5′UTR in both dark and light-grown seedlings [68, 70]. Unlike the promoter, the *εLCY* 5′UTR harboured several motifs reported to modulate transcription and post-transcriptional control over gene expression in plants [81]. For example, the most prominent *εLCY* 5′UTR fragment (TSS_2_) harboured an evolutionarily conserved viral IRES sequence and putative internal uORF along the stem of the central IRES loop within a centroid structural peak exhibiting a lower minimal entropy and fewer alternative secondary structure outcomes within this space of the mountain plot representation. Adjacent to the IRES nearest TSS_2_ was a region (CD-3) exhibiting low base-pairing probability and higher entropy indicative of alternative hairpin configurations. Viral IRES motifs can acquire structural definition changes in eukaryotes where they impact messenger RNA to recruit ribosomes, initiation factors, and/or RNA-binding proteins (RBPs) enabling cap-independent translational regulation of genes, particularly under stress conditions in response to environmental signals and/or chemical ligands [45, 71, 72, 82, 83]. In eukaryotes, an uORF in the 5′UTR could mediate ribosome stalling and translation attenuation yet RNA structural remodelling between hairpin definitions within CD-3 could facilitate formation of an active IRES. This control is reminiscent of translation attenuation in prokaryotic operons, where inhibition of translation elongation can regulate both mRNA translation and fine-tune gene expression by altering mRNA structure [50, 51, 84].

Does the native *εLCY* 5′UTR, uORF, or IRES alter promoter reporter activity or expression in planta? The native (unmodified) 5′UTR TSS_2_ fragment slightly enhanced FiLUC mRNA/activity ratio enabled by the CaMV35S promoter in leaves from three independent transgenic Arabidopsis lines. However, it was rather perplexing that the native 5′UTR TSS_2_ fragment did not affect transient FiLUC levels enabled by the CaMV35S promoter in tobacco leaves. The behaviour of the 35S-5′UTR::FiLUC transgene could vary in a heterologous host such as tobacco, due to the presence/absence of regulatory factors that can profoundly affect promoter-reporter activity in planta [85]. Nonetheless, deleting the small uORF and/or start codon (ATG) within the 5′UTR TSS_2_ fragment enhanced CaMV35S-enabled FiLUC levels in agrobacterium-infiltrated tobacco leaf tissues. Similarly, by removing the 3′ segment of the IRES (− 82 bp) including the uORF, CaMV35S-enabled transient FiLUC levels were also slightly enhanced in tobacco. This evidence indicates the native 5′UTR, including its uORF, can to some extent, influence reporter protein and/or mRNA levels enabled by the CaMV35S promoter. Given the uORF is positioned along the stem of the highly stable centroid peak loop within the conserved IRES, we propose that the native 5′UTR might need to acquire the alternative hairpin-3 structural definition within CD-3 to modulate promoter activity.

### Stabilisation of the *εLCY* 5'UTR RNA structural definition attenuates reporter activity

An unusual finding is reported in this study: *εLCY* 5′UTR structural shape variants repressed reporter levels. We do not have direct evidence to support conformational switching of the 5′UTR RNA structure. The native 5′UTR was dominated by 69% of RNA shape probabilities to form hairpin-1 and hairpin-2. In contrast, the hairpin-3 definition occupied by 29% of probabilities in the native 5′UTR, and when stabilised by introducing nine mutations within the *εLCY* 5′UTR, referred to as shape-1, it post-transcriptionally repressed foliar FiLUC activity in transient (tobacco) and transgenic (Arabidopsis) promoter-reporter expression systems. Shape-7 complemented seven of the nine mutations, leaving two mutations targeting the stem of hairpin-1/hairpin-2 within CD-3, and in the absence of these structural definitions, FiLUC activity was repressed without altering *FiLUC* transcript levels enabled by the CaMV35S promoter. The shape mutations not only impaired the formation of hairpin-1 and hairpin-2, but they also stabilised the probability of hairpin-3 in 85% of shape-7 structural definitions. The shape fragments showed negligible separation between the structure prediction curves in their mountain plot representations and generated a distal terminator-like structure. Deleting the sequence motif harbouring hairpin-1 did not impact FiLUC activity, suggesting the altered regulation was not due to the removal of a *cis*-acting motif. The post-transcriptional repression caused by shape-1/2/6/7 fragments could arise from the creation of a novel *cis*-acting element and/or stabilisation of the hairpin-3 RNA structural definition within CD-3 adjacent to the IRES. RBPs can assemble ribonucleoprotein complexes on RNA elements such as the IRES from the time of synthesis to degradation and modulate pre-mRNA processing, mRNA transcript stability, post-transcriptional, and translational regulation [82, 86, 87]. Hence, it is possible that the 5′UTR structural definition shape variants might impede or recruit a *trans*-acting RBP to modulate transcriptional and/or post-transcriptional processes, both of which are not exclusive, depending upon the light environment during seedling morphogenesis.

It remains perplexing why the shape variants, unlike the native 5′UTR, had such a profound effect on modulating promoter-reporter activity. The native 5′UTR and, to a greater extent, the shape-1 variant displayed higher FiLUC mRNA/activity ratios in transgenic Arabidopsis lines. This suggests that perhaps switching from a highly probable dominant (69%) to a less probable alternative (29%) RNA structural definition could underpin *εLCY* promoter regulation. While rare in plants, we speculate that the IRES or CD-3 hairpins could capture a signalling ligand and switch between the structural definitions. RNA structural bioinformatics identified the 5′UTR exhibited (1) a lower fit of greater separation between the structure prediction curves indicating alternative structural definitions surrounding three distinct hairpin/stem-loop structures within CD-3, (2) an optimal negative mfe range (− 21 to − 37 kcal/mol), (3) dot-bracket structural probabilities similar to 27 different classes of experimentally validated metabolite-binding RNA riboswitches, and (4) distinct and separate energy clouds reminiscent of an RNA structural switch. A typical RNA riboswitch harbours an aptamer domain that binds a ligand, and an expression platform that undergoes structural changes to regulate either initiation, elongation, or termination of transcription and/or translation [41, 43, 71]. Intriguingly, the *εLCY* IRES centroid structural peak had a lower minimal entropy with fewer secondary structure outcomes within this space and yet shared weak sequence homology with the SMK_box metabolite-binding RNA riboswitch. Collectively, in silico modelling of the *εLCY* 5′UTR revealed hallmark RNA regulatory features such as an IRES, uORF, and alternative structural definitions known to modulate transcription and/or translational processes in eukaryotes such as plants. Our favoured hypothesis is that the *εLCY* 5′UTR IRES and adjacent hairpin structures could constitute a new paradigm for ligand-captured switches that differ from metabolite-sensing riboswitches with regard to their small size, intrinsic stability, and definition of their structural conformational states [72].

### The stabilised 5'UTR RNA structural shape variant modulates feedback signalling

We tested the ability of the native 5′UTR and shape-1 fragments to modulate CaMV35S promoter activity in response to feedback signalling. NFZ treatment and hence inhibition of carotenoid biosynthesis in seedlings triggers retrograde signalling pathways that suppress *PhANG* expression and impair plastid biogenesis [78]. WT transgenic seedlings harbouring the native 5′UTR fused between the CaMV35S promoter and FiLUC reporter were unresponsive to NFZ, showing no change in FiLUC activity or transcript levels. Yet, dark- and light-grown WT transgenic seedlings harbouring the peculiar shape-1 variant displayed considerably reduced *FiLUC* activity levels in response to NFZ-induced signalling. Intriguingly, shape-1 also reduced *FiLUC* expression in de-etiolated but not etiolated seedlings, which mirrored changes in *εLCY* levels in planta. Therefore, the *εLCY* promoter and CaMV35S-5′UTR shape-1 transgene can be regulated by NFZ-induced feedback signalling at the transcriptional and post-transcriptional levels, depending upon the light environment during seedling morphogenesis.

Whether the feedback signalling was due to blocking biosynthesis of carotenoids, and perhaps an apocarotenoid and/or other retrograde signalling molecule, remains unclear. The loss-of-function in CRTISO activity impairs plastid biogenesis during *ccr2* seedling de-etiolation and triggers metabolic feedback signalling [10, 12, 61]. *ccr2* de-etiolated seedlings harbouring the 35S-shape-1::*FiLUC* transgene displayed lower reporter activity levels in comparison to WT lines, indicating the shape-1 fragment could harbour an aptamer domain responsive to feedback signalling. NFZ had a subtle effect to further reduce FiLUC activity in two independent *ccr2* de-etiolated seedlings, which is not surprising since NFZ and *ccr2* were reported to induce independent and yet overlapping feedback signalling pathways controlling *PhANG* expression in Arabidopsis [10, 12, 61]. The identity of a yet-to-be-identified retrograde signal, whether derived from *ccr2*-mediated inhibition of β-branch carotenoid biosynthesis (e.g., ACS) or generated by NFZ-induced signalling (e.g., Heme), and how its absence (or presence) mechanistically regulates the *εLCY* 5′UTR remains to be elucidated.

## Conclusions

The *εLCY* promoter including a prominent 5′UTR enabled reporter responsiveness mimicking changes in *εLCY* expression to genetic (*ccr2; carotenoid* c*hloroplast regulator 2*) and chemical (NFZ; inhibits cyclic carotenoid biosynthesis) perturbations during skoto- and photo-morphogenesis, linking retrograde signalling molecules to feedback between the plastid (etioplast and chloroplast) and nucleus [16] (Fig. 6). The native *εLCY* 5′UTR impacted the reporter mRNA/protein activity ratio. In silico modelling of the native *εLCY* 5′UTR revealed two distinct RNA structural probability definitions (69% vs. 29%) differing in their distal conserved domain-3, adjacent to IRES and uORF motifs—all of which are hallmarks of a 5′UTR expression platform [81, 88, 89]. Unlike the native 5′UTR, the variant shape-1 form stabilised the alternative hairpin-3 structural definition to resemble a terminator-like structure, and facilitated transcriptional, but also post-transcriptional regulation of the CaMV35S promoter in a light-dependent manner. The native 5′UTR and dominant structural definitions (hairpins-1/2) adjacent to the IRES may not be sufficient to mediate feedback regulation; however, stabilisation of the alternative hairpin-3 configuration in the peculiar shape variants renders the CD-3 and IRES expression platform responsive to NFZ and *ccr2*-mediated signalling. During plastid biogenesis, a signal could feedback to the nucleus and fine-tune *εLCY* mRNA/protein levels to modulate flux through the branch in the pathway and maintain carotenoid homeostasis (Fig. 6). Unravelling these regulatory mechanisms will facilitate biofortification strategies to challenge undesirable metabolic feedback observed when attempting to enhance health promoting β-branch carotenoids such as pro-vitamin A in crops.

## Methods

### Plant germplasm

All *Arabidopsis* germplasm are in the Columbia (Col-0) ecotype. Germplasm previously generated by other studies include: *ccr2.1*/*crtiso* [26], ziso#11C (*zic1-3*: Salk_136385) [90], *det1-1* (CS6158) [91], *det1-154* [10], *ccr2 det1-154* [10], *ccd4* (Salk_097984c), *ccr2 ccd4* [10], and MicroTom *tangerine*^*Mic*^ [92].

### Plant growth media, seed sterilisation, and chemical treatment assays

*Arabidopsis* seeds were stratified at 4–5 °C for 2–3 days to synchronise germination. Soil-grown *Arabidopsis* plants were sowed on DEBCO seed raising mix with Osmocote fertilizer (30 mL/5L) within growth cabinets (Climatron, Thermoline Scientific) or walk-in growth rooms fitted with fluorescent lights (120–150 μE m^−2^ s^−1^) and maintained under 16/8 h day/night photoperiod at 21 °C constant temperature. For artificial media-grown seedlings, seeds were first surface sterilised for 3–4 h by chlorine gas treatment (produced from mixing 100 mL 4% sodium hypochlorite and 3 mL of 37% w/v HCl), rinsed in 70% ethanol, followed by sterile H_2_O. *Arabidopsis* and tomato etiolated seeds were sown within a petri dish plate containing MS media (Caisson, MSPO1) supplemented with 0.5 × Gamborg’s vitamin solution (0.5 mL/L) (Sigma-Aldrich), and 0.5% phytagel (Sigma-Aldrich) (5 g/L). Plates were wrapped in a double-layered aluminium foil and incubated in a dark growth cabinet at 21 °C and 60% constant humidity for 7 days after stratification. For de-etiolation experiments, *Arabidopsis* seeds were stratified and grown in the dark at 21 °C for 4 days prior to exposed seedlings to constant low fluorescent lighting (80–90 μE m^−2^ s^−1^) for 72 h at 21 °C, unless otherwise indicated. Etiolated tissues were harvested under a non-photosynthetic dim green LED light in a dark room. NFZ treatment of seedlings was performed by growing seedlings in MS media supplemented with 5 μM NFZ (dissolved in 100% DMSO).

### Molecular vector construction

pT*εLCY*Prom-*εLCY*5′UTR::FiLUC (previously referred to as pT*eLCY*−4F::FiLUC) harbours a − 450-bp region (promoter plus 5′UTR) cloned upstream of the *FiLUC* gene into pTm35:FiLUC (digested with *Xba*I/*Nco*I to remove the minimal 35S promoter), as previously described [62]. The − 133 bp *εLCY* 5′UTR and series of *εLCY* 5′UTR mutated and deleted (MutDels) sequence variants downstream of a min35S domain that incorporated 5' *Xho*I and 3' *Nco*I restriction sites were synthesised and cloned into the pMat intermediate plasmid (Life Technologies, Germany). Fragments were subsequently removed from pMat and cloned into pT35enh::*FiLUC* binary vector using *Nco*I/*Xho*I restriction endonuclease digestion and T4 ligation as previously described [93]. This generated eight MutDels constructs where the *εLCY* 5′UTR was fused downstream of the CaMV35 promoter and upstream of the luciferase reporter gene (Fig. 4A, Additional file 1: Figure S7D).

The pT*35S*-*εLCY*5′UTR-Shape1::FiLUC vector was further modified by sequentially restoring mutated sequences to generate three new constructs referred to as “Mut2Y” (Shape-2), “Mut6Y” (Shape-6), and “Mut7Y” (Shape-7) (Additional file 1: Figure S7D). The subsequent new destination vector was modified such that *Hind*III sites flanking the 35Senh and min35S domain were replaced to introduce an internal *XbaI* site after the min35S domain and internal to the *Hind*III site. In brief, the region between 35Smin and *εLCY* 5′UTR insert was amplified using Platinum® Pfx DNA polymerase (Invitrogen) with HindIII_T35_F1 and HindIII + XbaI_T35_R1 primers and the amplicon cloned into a pCR4-TOPO intermediate vector. pCR4-TOPO::35Smin::*εLCY*5′UTR was digested with *HindIII*, and the purified T35Smin-*εLCY*5′UTR fragment was ligated into the pT35S-*εLCY*5′UTR::FiLUC vector digested with *HindIII* and treated with FastAP Thermosensitive Alkaline Phosphatase (1 U/μL) (ThermoFisher) to create pT35SX-*εLCY*5′UTR::FiLUC. The new MutDels of the *εLCY*5′UTR fragments were amplified using 1F_Mut5UTR, 4 F Mut5UTR, 6F_Mut5UTR plus 1R_Mut5UTR primers (harbour flanking 5' *XbaI* and 3' *NcoI* sites) and pT35S-*εLCY*5′UTR::*FiLUC* as DNA template to generate amplicons for Mut2Y, Mut6Y, and Mut7Y, respectively. All amplicons were cloned into pCR®4-TOPO® and validated by Sanger sequencing. Mut2Y, Mut6Y, and Mut7Y amplicons were subsequently digested from the intermediate vector and ligated into the modified pT35SX-*εLCY*5′UTR::FiLUC vector to create pT35S-εLCY5′UTR-Mut2Y::FiLUC, pT35S::εLCY5′UTR-Mut6Y::FiLUC, and pTV35S-εLCY5′UTR-Mut7Y::FiLUC binary constructs using an *Xba*I/*Nco*I cloning strategy.

The bacterial CrtI gene from *Pantoea agglomerans* (*PaCrtI*) was fused downstream of the *Arabidopsis* small subunit (SSU) of RuBisCo transit peptide (56 amino acids) [55] controlled by the CaMV35S enhancer to create the binary vector pT35S::*PaCrtI*. The nopaline synthase terminator (NosT) within pT35enh::*FiLUC* [62] was re-amplified to include additional 5′ (*Bgl*II and *Avr*II) and 3′ (*Pst*I) restriction sites, cloned into the TOPO-Blunt-II intermediate vector (Invitrogen), and subsequently used to replace the original NosT to create pT35enh::*FiLUC-*NosTMod. The SSU-*PaCrtI* gene was chemically synthesized and incorporated bordering '5' (*Nco*I) and 3′ (*Avr*II) restriction sites cloned into the pMat intermediate plasmid (Life Technologies, Germany). The *FiLUC* gene was excised from pT35enh::*FiLUC-*NosTMod using *Nco*I/*Avr*II and replaced with *PaCrtI* to create the pT35enh::SSU-*PaCrtI* binary vector.

### Plant transformation and selection of transgenic lines

*Arabidopsis* transformation with Agrobacterium harbouring binary plasmids was performed using a modified floral dip method [94]. Agrobacterium and the containing binary plasmid were selected on Rifampicin (25 µg/L) and spectinomycin (100 µg/L). The TDNA within the binary vectors harboured *PHOSPHINOTHRICIN ACETYL TRANSFERASE* (*BAR*) that allowed the selection of transgenic and homozygous lines by spraying plants with BASTA herbicide (50 mg/L) as previously described [95]. The generation of pTm35enh::*FiLUC* transgenic WT lines was previously described [62]. For exogenous expression of *PaCrtI* in *ccr2*, independent transgenic lines haboring T35enh::*SSU-PaCrtI* were selected by the presence of their T-DNA insertion (e.g., resistance to BASTA herbicide) and functionality of *PaCrtI* (e.g., displayed green leaves when grown on MS media containing 2.5 µM NFZ). Representative homozygous lines that displayed a green phenotype and segregated in a typical 3:1 Mendelian manner (chi-squared test) was chosen for further analysis.

### *E. coli* transformation, electroporation, and tobacco agro-infiltration

Chemically competent *E. coli* (DH5α) cells were prepared as previously described [96]. Plasmids were transferred into competent *E. coli* cells using a conventional heat-shock (42 °C) transformation method. Electrocompetent Agrobacterium (LBA4404) cells were prepared as previously described [97]. Plasmids were electroporated into electro-competent Agrobacterium using a Bio-Rad MicroPulser (Bio-Rad, Hercules, USA) according to the manufacturer’s protocol. Agro-inoculation of tobacco leaves was performed by resuspending a bacterial culture (growing in the exponential phase) in infiltration media (10 mM MgCl_2_, 100 μM acetosyringone and 1 mM MES; 2-[N morpholino] ethanesulfonic acid; pH 5.6) to an OD_600_ ~ 0.6 and injected into young tobacco leaves via a needless syringe as previously described [95].

### Luciferase bioassays

Luciferase bioassays were performed as described [98], and used to select representative transgenic lines as well as distinguish between homozygous and heterozygous lines based upon luminescence (*FiLUC* activity) emitted from identical-sized leaf disks collected from mature leaves of independent plants. Individual leaf disks were placed on 35 μL assay media (50 mM MES (pH 5.6), 0.5% glucose, 2 mM NaPO_4_, 0.5% v/v DMSO) in individual wells of a 96-well plate (Corning® NBS). Then 15 μL of luciferin assay media (1 mM luciferin, 50 mM MES (pH 5.6), 0.5% glucose, 2 mM NaPO_4_, 0.5% v/v DMSO) was added prior to quantifying luminescence as previously described [93] using the CLARIOstar® *Plus* plate reader (BMG LABTECH). Luminescence imaging of whole plants was performed by incubating them in 0.5 mM luciferin for 10 min prior to imaging light emission using a CCD camera (Model DV435; Andor Technologies) and ImagePro Plus 4.5.1 software (Media Cybernatics). Captured images of luminescence were enhanced and overlayed using NIH ImageJ software (version 1.53).

### Total RNA isolation and cDNA synthesis

Frozen leaf samples were physically disrupted using TissueLyser (Qiagen). The total RNA was extracted from the fine powder of plant tissue with Spectrum™ Plant Total RNA kit (Sigma-Aldrich) according to the manufacturer’s protocol. Isolated total RNA samples were quantified with NanoDrop 2000 and used for cDNA synthesis after validating their integrity on a 1% TBE agarose gel. The total RNA (1 to 2 μg) was used to synthesise cDNA with the Bioline Tetro cDNA synthesis kit by following the manufacturer’s instructions. The reaction mix was prepared with 1 μL Oligo (dT), 1 μL 10 mM dNTP mix, 4 μL 5 × RT Buffer, 1 μL RiboSafe RNase Inhibitor, 1 μL Tetro Reverse Transcriptase (200 U/μL), and 1–2 μg total RNA and DEPC-treated dH_2_O up to 20 μL total reaction volume. The mixture was incubated at 45 °C for 30 min, then at 85 °C for 5 min to terminate and chilled on ice for 5 min.

### Quantification of relative gene expression and PCR

The relative transcript abundance in plant tissues was quantified using LightCycler 480 (Roche, Australia). Primers were designed according to the sequence information of organisms in the NCBI GenBank and TAIR databases using Primer3Plus [99]. Quantitative reverse transcription PCR (qRT-PCR) was performed with the mixture of 2 μL of primer mix (2 μM from each F & R primer), 1 μL 1/10 diluted cDNA template, 5 μL LightCycler 480 SYBR Green I Master mix and distilled water up to the total volume of 10 μL. For each sample, three technical replicates for each of one to three biological replicates were tested. The relative gene expression levels were calculated by using relative quantification (Target Eff ^Ct(Wt−target)^/Reference Eff ^Ct(Wt−target)^) and fit point analysis [100]. The *Protein Phosphatase 2A* (*PP2A*, At1g13320) and/or *TIP41* (At4g34270) were used as house-keeper reference control genes for all qRT-PCR experiments [101]. All primer sequences are listed in Additional file 2: Table S3.

General PCR reactions were performed by preparing a mixture with 2.5 μL 10 X Taq polymerase buffer, 1 μL dNTP (10 mM), 2 μL MgCl_2_ (10 mM), 0.25 μl Taq DNA polymerase (5 U/μL), 1 μL/each of forward (F) and reverse (R) primers (10 μM/μL), 1 μL template DNA (~ 100 ng/μL), and dH_2_O up to 25 μL. Thermal-cycler adjustments were as follows: 5 min 95 °C pre-denaturation followed by 35 cycles of 1 min at 95 °C, 1 min at 45–65 °C 1 min at 72 °C and 5 min for the final extension at 72 °C. The annealing temperature was variable depending on the used primers. Two percent TBE agarose gel was used to confirm the length of each reaction product.

### 5′ Rapid Amplification of cDNA Ends (5′RACE)

The integrity of total RNA samples used for Rapid Amplification of cDNA Ends (RACE) assays was validated using Agilent 2100 Bioanalyzer in WSU’s NGN facility. 5′RACE assays were performed using the GeneRACER kit according to the manufacturer’s protocol. RACE cDNA libraries were prepared with SuperScript III Reverse Transcriptase using GeneRacer™ Oligo dT Primer according to the manufacturer’s protocol. 5′ ends of gene-specific cDNAs were amplified with Platinum® Pfx DNA polymerase (Invitrogen) and subsequently cloned into pCR®4-TOPO® for Sanger sequencing. The sequences of gene-specific primers used in the first step of amplification and the nested PCR can be found in Additional file 2: Table S3.

#### Carotenoid purification and HPLC analysis

Frozen tissues were homogenised to a fine powder using TissueLyser® (QIAGEN), while root tissues, on the other hand, were homogenised using mortar and pestle. Carotenoids were extracted as previously described [102] using a reverse-phase high-performance liquid chromatography (HPLC) (Agilent 1260 Infinity) and a YMC-C30 (250 × 4.6 mm, S-5 µm) column. Carotenoids and chlorophylls were identified based on their retention time relative to known standards and their light emission absorbance spectra at 440 nm (chlorophyll, β-carotene, xanthophylls, pro-neurosporene, tetra-*cis*-lycopene, other neurosporene and lycopene isomers), 400 nm (ζ-carotenes), 340 nm (phytofluene), and 286 nm (phytoene). Absolute quantification of carotenoid and chlorophyll pigments was performed as described [103].

### In silico bioinformatics analysis

In silico prediction of RNA secondary structure formations was performed using desktop and online versions of RNAShapes (https://bibiserv.cebitec.uni-bielefeld.de/rnashapes) [75, 104] and RNAfold (Vienna package; http://rna.tbi.univie.ac.at/cgi-bin/RNAWebSuite/RNAfold.cgi) [76, 105]. UTRscan (http://bioinformatics.ba.itb.cnr.it/?Software___UTRscan) and IRESite (https://iresite.org) were used to assess sequence homology against a UTR database and predict functional elements and validate against a database of experimentally verified IRES [106, 107]. cis-acting regulatory elements/motifs were identified in the − 450-bp *εLCY* promoter region using PlantCare [108]. NCBI Blast (https://blast.ncbi.nlm.nih.gov/Blast.cgi) and Primer3Plus (version 2.4.0) (https://primer3plus.com) [99] were used to identify gene sequences and to design qPCR primers, respectively. Geneious (version 10.2.6) was used to perform the sequence alignments. CLC Sequence Viewer (version 8.0.0) in silico cloning editor was used to design the plasmids. Genevestigator was used to interrogate the effect of NFZ on gene expression in WT seedlings grown under light [109]. *Arabidopsis* eFP browser was used to interrogate the differential expression of *εLCY* in young and mature leaves, using data from a previous study [110, 111]. Statistical analysis for qPCR and HPLC results were performed with SigmaPlot (v14.0) using one-way or two-way ANOVA with post hoc Tukey (HSD), depending on the experimental setup. Figures were designed using commercial software Adobe Illustrator.

## Supplementary Information

Additional file 1: Figure S1. Acyclic *cis*-carotene and cyclic carotenoid biosynthesis in Arabidopsis. 15-*cis*-phytoene is synthesised from the condensation of geranylgeranyl pyrophosphate by PSY, which is the first rate-limiting step in linear *cis*-carotene biosynthesis. Next, 15-*cis*-phytoene and 9,15-di-*cis*-phytofluene undergo desaturation by PDS to generate 9,15,9′-tri-*cis*-ζ-carotene. The enzymatic activity of PDS can be blocked by the chemical inhibitor norflurazon (NFZ). Isomerization of 9,15,9′-tri-*cis*-ζ-carotene to 9,9′-di-*cis*-ζ-carotene is catalysed by Z-ISO, a reaction also facilitated by photoisomerization. ZDS catalyses the production of 7,9,9′,7′-tetra-*cis*-lycopene (prolycopene) from 7,9,9′-tri-*cis*-neurosporene. CRTISO catalyses the final isomerization of 7,9,9′,7′-tetra-*cis*-lycopene to all-*trans*-lycopene (lycopene), a reaction also fulfilled by light-mediated photoisomerization. The loss-of-function *ziso* and *ccr2* mutant seedlings (red font) accumulate linear *cis*-carotenes, block cyclic carotenoid accumulation, impair plastid biogenesis, and trigger feedback signalling in etiolated seedlings. The pathway then bifurcates after lycopene to produce downstream cyclic carotenoids (closed carbon ring structures at the ends). εLCY mediates the cyclization of all-*trans*-lycopene (acyclic carotenoid having linear carbon double bonds in the trans-configuration) into a temporary intermediate α-carotene that becomes rapidly hydroxylated to produce lutein. βLCY modulates the cyclization of all-*trans*-lycopene into β-carotene. The loss-of-function *ccd4* mutant seedling (red font) impairs the cleavage of β-carotene into β-apocarotenoids. Hydroxylation of β-carotene by βOHase next produces zeaxanthin that is readily transformed into violaxanthin via antheraxanthin, incorporating the 5,6-epoxy group into the 3-hydroxy β-rings by ZEP. VDE facilitates the de-epoxidation process in two stages, converting violaxanthin back into zeaxanthin, a reversible reaction effective at dissipating excess excitation energy. The reversible interconversion referred to as the xanthophyll cycle is crucial for non-photochemical quenching (NPQ) and plant acclimation to light level fluctuations. The final stage of carotenoid biosynthesis involves the transformation of violaxanthin into neoxanthin through the enzymatic activity of NXS. Yellow and red highlighted areas reveal the acyclic *cis*-carotenes (linear carbon configuration without any ring structures) and cyclic carotenoids (closed carbon ring structures at the ends), respectively either side of all-*trans*-lycopene. Abbreviations: PSY, PHYTOENE SYNTHASE; PDS, PHYTOENE DESATURASE; Z-ISO, ζ-CAROTENE ISOMERASE; ZDS, ζ-CAROTENE DESATURASE; CRTISO, CAROTENOID ISOMERASE; CCD4, CAROTENOID CLEAVAGE DIOXYGENASE 4; εLCY, LYCOPENE ε-CYCLASE; βLCY, LYCOPENE β-CYCLASE; β-OHase, non‐heme di‐iron β-CAROTENE-3-HYDROXYLASE; ZEAXANTHIN EPOXIDASE; ZEP, VIOLAXANTHIN DE-EPOXIDASE; VDE, NEOXANTHIN SYNTHASE; NSY, BACTERIAL CAROTENOID ISOMERASE; CRTI (also referred to as *Pantoea ananatis* PHYTOENE DESATURASE), SDG8; SET DOMAIN GROUP 8, *ccr2*; carotenoid chloroplast regulator 2, *ccr1*; carotenoid chloroplast regulator 1. Figure S2. Absolute levels of linear *cis*-carotenes in *ziso*, *ccr2 ziso*, and *ccr2* etiolated tissues exogenously expressing *PaCrtI*. (A) Linear *cis*-carotene levels in *ccr2*, *ziso*, and *ccr2 ziso* etiolated seedlings. (B) Linear *cis*-carotenes and (C) cyclic carotenoids in etiolated tissues of *ccr2* and *ccr2* T35enh::SSU-*PaCrtI*#11. Significant differences are denoted by lettering (Two-way ANOVA) or stars (one-way ANOVA; *p <0.05, **p <0.005, ***p <0.001). Data are representative of three separate experiments, and bars define standard error (*n*=3-4). Abbreviations: violaxanthin (Vio), neoxanthin (Neo), antheraxanthin (Ant), lutein (Lut), β-carotene (β-car), pro-neurosporene (P-Neu), prolycopene (P-Lyc), 9,15,9’-tri-*cis*-ζ-carotene (tc-ζc), 9,9’-di-*cis*-ζ-carotene (dc-ζc), 15-*cis*-phytofluene (Phf), 15-*cis*-phytoene (Phy). Figure S3. Loss-of-function in CCD4 enhances β-carotenoid accumulation in etiolated WT and *ccr2* seedlings. Quantification of carotenoids in dark-grown seedlings. A) Cyclic carotenoid levels in WT and *ccd4* etiolated cotyledons. B) Linear *cis*-carotene levels in WT and *ccr2 ccd4* etiolated cotyledons. Significant differences are denoted by stars (ANOVA; *p <0.05, **p <0.001). Data represents multiple experiments, and bars denote standard error (*n*=3-4). Abbreviations: Violaxanthin (Vio), Neoxanthin (Neo), Antheraxanthin (Ant), Zeaxanthin (Zea), Lutein (Lut), β-carotene (β-car), pro-neurosporene (P-Neu), prolycopene (P-Lyc), 9,15,9’-tri-*cis*-ζ-carotene (tc-ζc), 9,9’-di-*cis*-ζ-carotene (dc-ζc), 15-*cis*-phytofluene (Phf), 15-*cis*-phytoene (Phy). Figure S4. Carotenoid content in norflurazon-treated wild-type and *ccr2* etiolated seedlings. A-B) HPLC chromatogram showing 440nm carotenoid traces (A) and absolute levels of phytoene (A286nm) as well as phytofluene (A348nm) (B) from WT and* ccr2* dark-grown seedlings grown on artificial media with (+) or without norflurazon (NFZ; 5 µM). Significant differences are denoted by lettering (Two-way ANOVA). C) Representative image of a norflurazon (NFZ; 5 µM) treated and untreated (control) wild-type (WT) seedling. Data represent three separate experiments, and standard error bars are displayed (*n*=3-4). Abbreviations: violaxanthin (V), neoxanthin (N), antheraxanthin (A), lutein (L), zeaxanthin (Z), β-carotene (βc), pro-neurosporene (P-Neu), prolycopene (P-Lyc), 9,15,9’-tri-*cis*-ζ-carotene (tc-ζcar), 9,9’-di-*cis*-ζ-carotene (dc-ζcar),. Figure S5. Pigment content and *εLCY* expression in young and old leaves. A) Carotenoid levels in WT and *ccr2* de-etiolated seedlings following 72 hrs of constant illumination. B) Absolute expression *εLCY* levels in young and older mature leaf tissues as depicted by the TAIR eFP browser (Klepikova Atlas). C) Carotenoid and chlorophyll levels in old mature and young emerging leaves from the 3-weeks-old rosette leaves. Data are representative of three separate experiments, and bars define the standard error of the mean (*n*=3-4). Significant differences are denoted by lettering (Two-way ANOVA) or stars (one-way ANOVA; *p <0.05, **p <0.005, ***p <0.001). Abbreviations: violaxanthin (Vio), neoxanthin (Neo), antheraxanthin (Ant), lutein (Lut), β-carotene (β-car), chlorophyll a (Chla), chlorophyll b (Chlb). Figure S6. Luciferase activity and expression in plant tissues from independent transgenic lines harboring promoter-reporter gene fusions. A-B) Independent transgenic lines harbouring *εLCY*Prom-*εLCY*5′UTR::*FiLUC* (εLP::*FiLUC*) and CaMV35S::*FiLUC *(35S::*FiLUC*) (B) promoter-reporter transgene fusions. Luciferase (FiLUC) activity was quantified as the relative light units (RLU) emitted from equal-sized leaf discs (two leaf discs per leaf/plant) dissected from independent heterozygous transgenic plants (T2) using the in vivo floating leaf-disk bioassay [91]. Independent lines are ordered by increasing luciferase activity along the x-axis and representative lines for (A) εLCY (εLP#2e; εLP#4c) and (B) CaMV35S (35S#B30, 35S#C1) are indicated. C) Average relative lights units (RLU) emitted by leaves from homozygous transgenic Arabidopsis lines harbouring 35S::*FiLUC* (35S), 35S-5′UTR::*FiLUC* (5′UTR), and 35S-shape1::*FiLUC* (Shape-1) promoter-reporter gene fusions. Standard errors bars are displayed (*n*=4-10; two discs/leaf from multiple leaves and plants), The average RLU for multiple independent transgenic lines with standard error bars are shown (*n*=4; 35S, *n*=11; 5′UTR, *n*=15; Shape-1). Figure S7. Deletions, mutations, *cis*-acting motifs and conserved domains in the ε*LCY* promoter and 5′UTR. A) Original, uncropped gel blot image displaying PCR amplicons from two independent 5′RACE (Rapid Amplification of cDNA Ends) experiments using etiolated (dark) and de-etiolated (light) tissues to extract *εLCY* mRNA. A 50bp DNA ladder was used as the size marker. Sequencing PCR products revealed three amplicons having homology to the *εLCY* upstream region. B-C) *cis*-acting regulatory elements and conserved domains positions within the -450 bp *εLCY* promoter region identified using PlantCare [106], UTRScan and IRESite [104, 110] bioinformatics software. The putative elements, Downstream Promoter Element (DPE), Intron-Mediated Enhancer (IME), Initiator (Inr), and Internal Ribosome Entry Site (IRES) elements were identified by aligning the *εLCY* promoter sequence using Clustal Omega alignment software. Transcription Start Sites (TSS) and conserved domains are marked upstream of the start codon (ATG). An upstream open reading frame (uORF) consisting of 2 amino acids (Met, Val) is denoted by a red underline (-47 to -39 bp). Nucleotide positions shown refer to the length of *εLCY *5′UTR fragments. D) Sequence alignment of *εLCY* 5′UTR mutation and deletion (MutDels) fragment variants. Alignment was performed using Geneious Prime Software (v10.2.6). The sequence logo shows the mutations introduced. The font size reflects the abundance of the nucleotide. Nucleotide positions indicated above the sequence logo reveal the position of relevant MutDels. The red vertical box signifies the two mutated nucleotides in shape fragments that alter 5′UTR RNA structural definitions and CaMV35S promoter-enabled reporter activities. Figure S8. Representative RNA secondary structural plots and prediction probabilities. Vienna RNA structural representations generated by RNAShapes using dot-bracket notation for the* εLCY* 5′UTR (A), shape-1 (B), shape-2 (C), shape-6 (D), and shape-7 (E) sequences. Red lines and circled DNA base pairs denote the hairpin-1, hairpin-2 and hairpin-3 structural definitions in the *εLCY* 5′UTR (A). The percentage (%) value displayed above structural definitions reflects the highest percentage shape probability differentiating the dot-bracket notations by hairpin definitions with CD-3 (Figure S9). Figure S9. In -silico RNA structural analysis of the *εLCY* shape variants modulating a post-transcriptional expression platform. (A) RNAfold mountain plots showing RNA secondary structure predictions for *εLCY* 5'UTR, shape-1, shape-2, shape-6, and shape-7. Plots represent the mfe structure, thermodynamic ensemble of RNA structures (pf), and the centroid structure in a plot of height m(k) versus position (bp), where the height is given by the number of base pairs enclosing the base at a position. "mfe" represents minimum free energy structure; "pf" indicates partition function; "centroid" represents the best average structure. Loops correspond to plateaus (hairpin loops are peaks), and helices to slopes. RNAfold calculates the entropy of each bp along the RNA sequence, indicating the stability of the RNA secondary structure per given sequence (starting at -1 to -133 bp) which is shown below the mountain plot. Low entropy values indicate high stability of RNA secondary structures indicated by the mountain height/bp. (B) RNAShapes analysis of the 5′UTR, shape-1, shape-2, shape-6, and shape-7 sequences showing Vienna shape representatives (dot-bracket notation), percentage shape probability (Prob.) and minimal free-energy (MFE; kcal/mol) associated with secondary structure formation [73, 102]. Dot-bracket notation for RNA secondary structures is represented by a string length of matching brackets and dots, where a base pairing is represented by a '(' and unpaired bases are represented by dots '.' [75]. Green or red dot-brackets denote similar structural representations. The structural probability (prob.) >0.01 (1%) and minimum free energy (MFE) are displayed. Red and orange lines above the *A. thaliana* 5′UTR sequence denote CD-3 and IRES motif, respectively. The boxed regions denote three stem-loop hairpin structures found in Conserved Domain-3 (CD-3). (C) Base-pairing probability and (D) positional entropy of the most probable structures for *εLCY* 5'UTR, shape-1, shape-2, shape-6, and shape-7 as generated by RNAfold software using default settings. A base pairing probability value of 1 means it is highly stable. A positional entropy value of 0 indicates the base-pair is always unpaired or paired with its partner.

Additional file 2: Table S1. Correlations between the regulation of *εLCY* and carotenoid levels in plants. Table S2. Carotenoid percent composition in rosette leaf tissues from *ccr2* lines harboring 35Senh::SSU-*PaCrtI*. Table S3. Primers used for qPCR, cloning, and 5'Rapid Amplification of cDNA Ends.

Additional file 3: Dataset S1. Nucleic acid sequence-based homology analysis of *εLCY* 5′UTR for screening similarity with Rfam riboswitch database.

Additional file 4: Dataset S2. The shape-switching potential of 27 different riboswitch classes found in the Rfam database. Analysis was performed based on RNA structures with minimum free energy and maximum stability. The number of shapes with >10% minimum probability is considered significant.

## Data Availability

Not applicable.

## Abbreviations

ACS: Apocarotenoid signal
β-ACS: β-Carotene-derived apocarotenoid signal
*cis*-ACS: *cis*-Carotene-derived apocarotenoid signal
FiLUC: Intron modified *FIREFLY* (*Photinus pyralis*) *LUCIFERASE* gene
MutDels: Mutations and/or deletions
NFZ: Norflurazon
Nos^t^: Nopaline synthase terminator
uORF: Upstream open reading frame
RLU: Relative light units
TSS: Transcription start site
5′UTR: 5′ Untranslated region
