# Supplementary material for: Plastid-mediated feedback regulation of Arabidopsis LYCOPENE EPSILON CYCLASE is modulated by the promoter and a 5′UTR structural variant harbouring a conserved IRES
Source: BMC Biol. 2025 Nov 4;23:334. doi: 10.1186/s12915-025-02436-z (PMC12584516; doi:10.1186/s12915-025-02436-z)
Supplement: Supplementary file 1 — Additional file 1: Figure S1. Acyclic cis-carotene and cyclic carotenoid biosynthesis in Arabidopsis. 15-cis-phytoene is synthesised from the condensation of geranylgeranyl pyrophosphate by PSY, which is the first rate-limiting step in linear cis-carotene biosynthesis. Next, 15-cis-phytoene and 9,15-di-cis-phytofluene undergo desaturation by PDS to generate 9,15,9′-tri-cis-ζ-carotene. The enzymatic activity of PDS can be blocked by the chemical inhibitor norflurazon (NFZ). Isomerization of 9,15,9′-tri-cis-ζ-carotene to 9,9′-di-cis-ζ-carotene is catalysed by Z-ISO, a reaction also facilitated by photoisomerization. ZDS catalyses the production of 7,9,9′,7′-tetra-cis-lycopene (prolycopene) from 7,9,9′-tri-cis-neurosporene. CRTISO catalyses the final isomerization of 7,9,9′,7′-tetra-cis-lycopene to all-trans-lycopene (lycopene), a reaction also fulfilled by light-mediated photoisomerization. The loss-of-function ziso and ccr2 mutant seedlings (red font) accumulate linear cis-carotenes, block cyclic carotenoid accumulation, impair plastid biogenesis, and trigger feedback signalling in etiolated seedlings. The pathway then bifurcates after lycopene to produce downstream cyclic carotenoids (closed carbon ring structures at the ends). εLCY mediates the cyclization of all-trans-lycopene (acyclic carotenoid having linear carbon double bonds in the trans-configuration) into a temporary intermediate α-carotene that becomes rapidly hydroxylated to produce lutein. βLCY modulates the cyclization of all-trans-lycopene into β-carotene. The loss-of-function ccd4 mutant seedling (red font) impairs the cleavage of β-carotene into β-apocarotenoids. Hydroxylation of β-carotene by βOHase next produces zeaxanthin that is readily transformed into violaxanthin via antheraxanthin, incorporating the 5,6-epoxy group into the 3-hydroxy β-rings by ZEP. VDE facilitates the de-epoxidation process in two stages, converting violaxanthin back into zeaxanthin, a reversible reaction effective at dissip [file 12915_2025_2436_MOESM1_ESM.pdf]

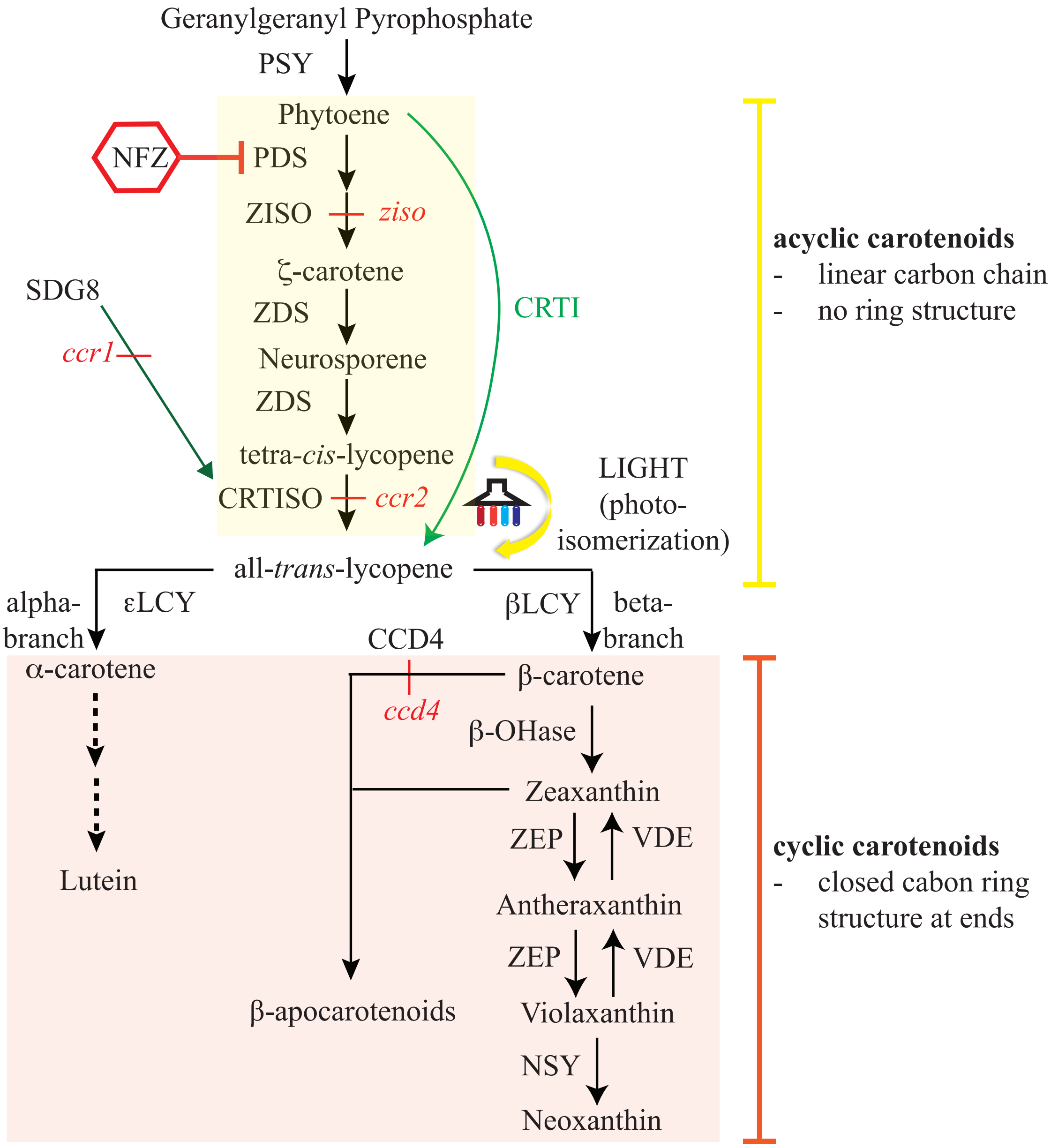

Figure S1. Linear *cis*-carotene and cyclic carotenoid biosynthesis in Arabidopsis

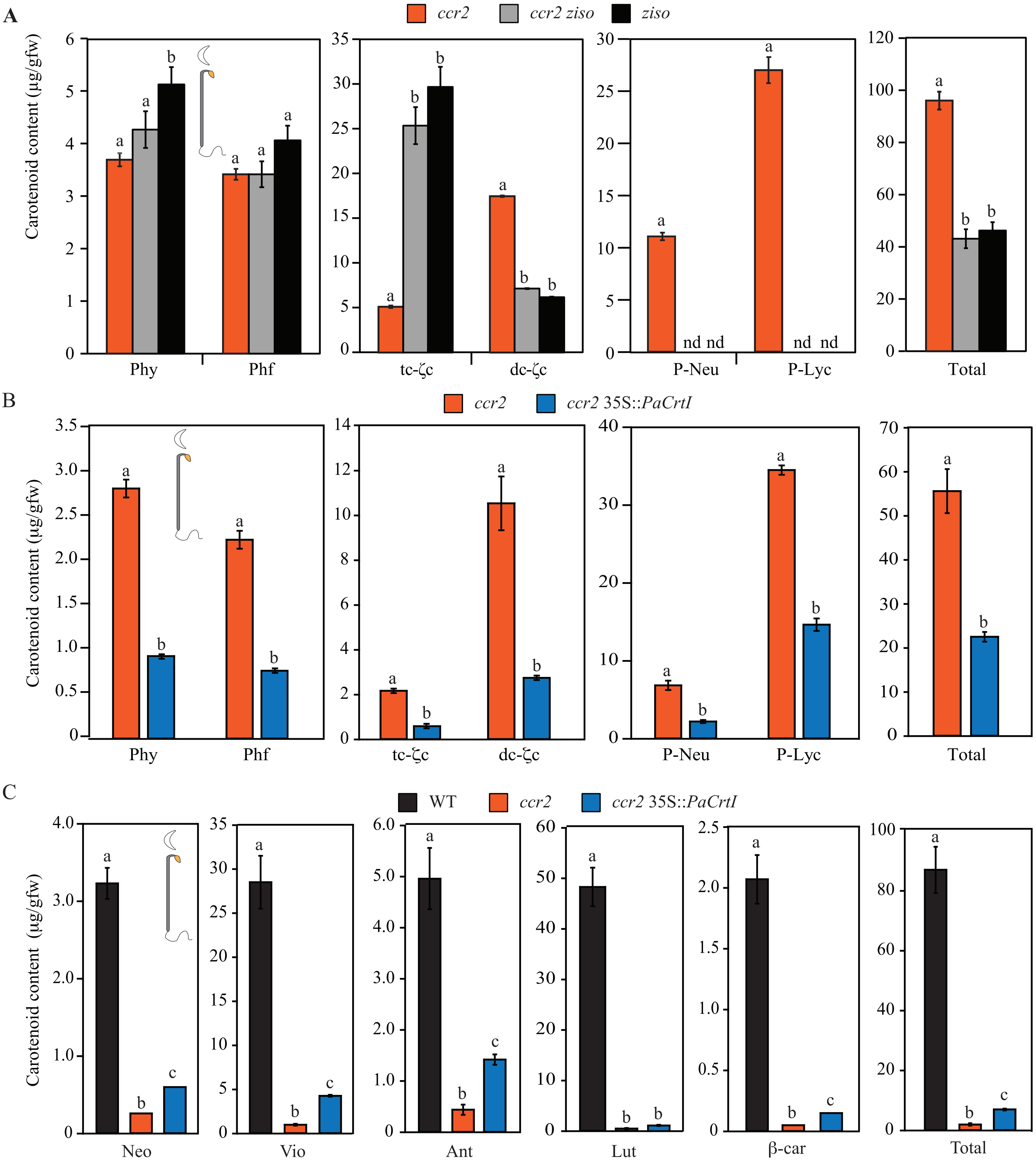

Figure S2. Abolute levels of acyclic linear *cis*-carotenes in *ziso*, *ccr2 ziso*, and *ccr2* etiolated tissues exogenously expressing *PaCrtI*.

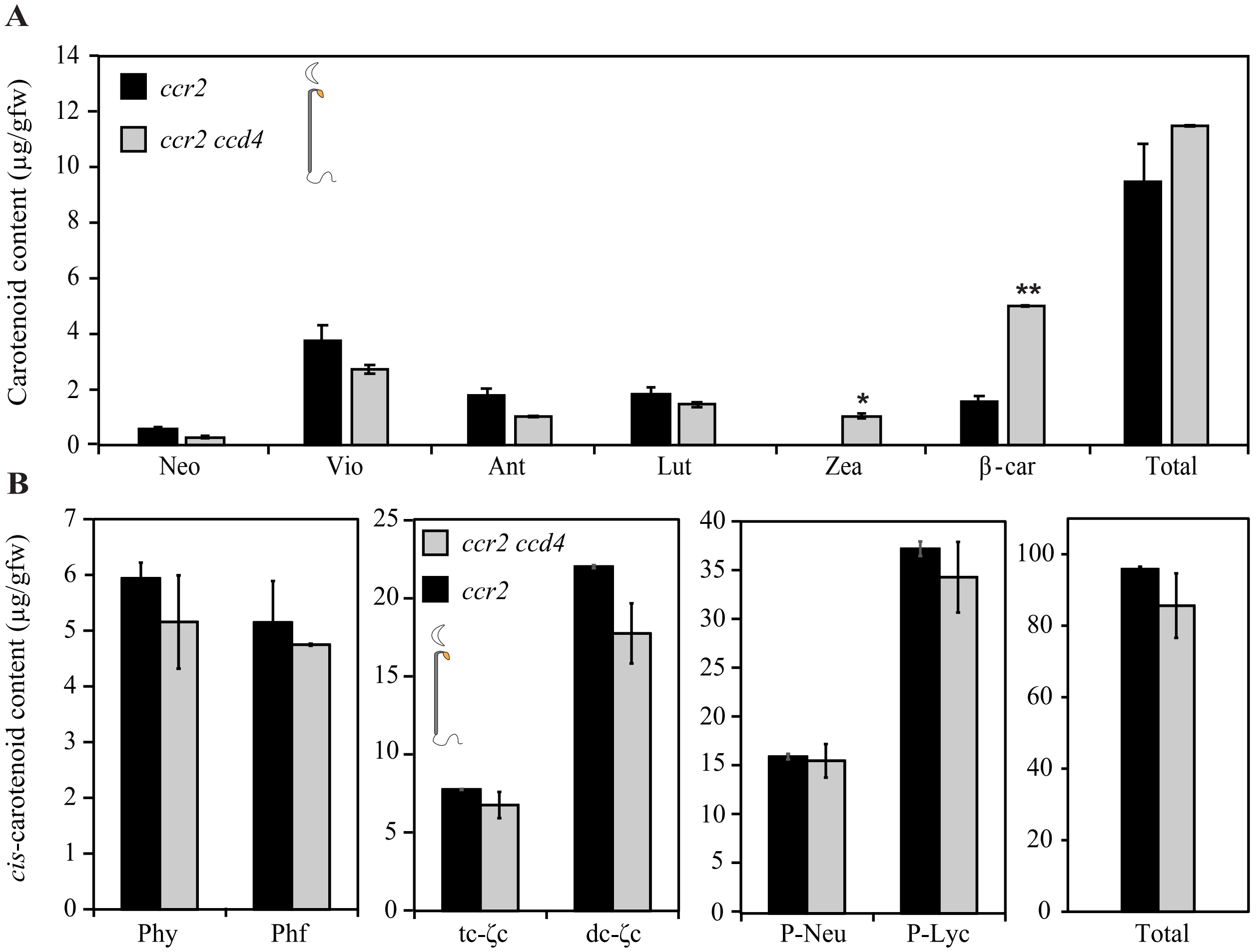

Figure S3. Loss-of-function in CCD4 enhances β-carotene and zeaxanthin accumulation in *ccr2* etiolated seedlings.

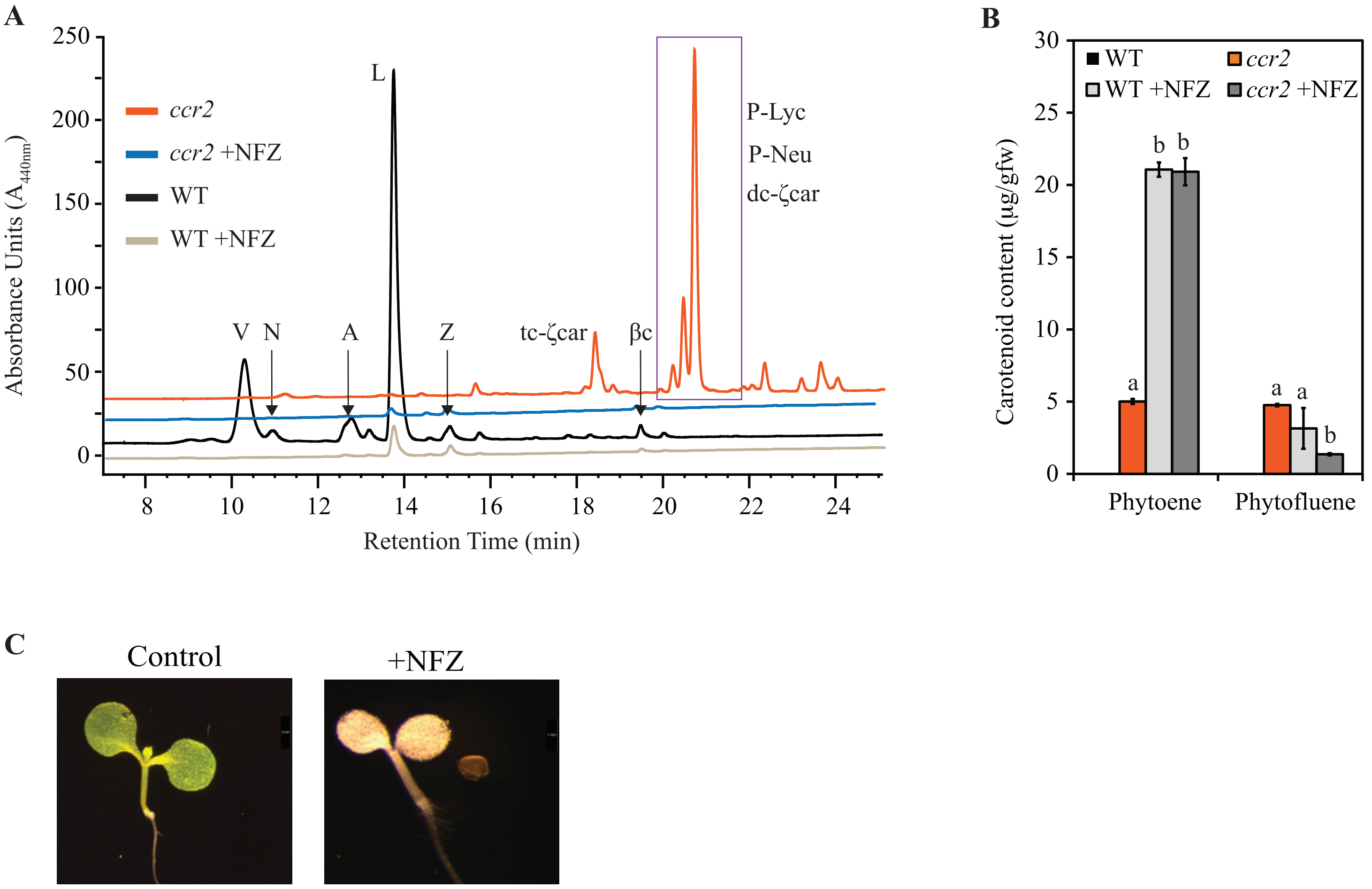

Figure S4. Carotenoid content in norflurazon-treated wild-type and *ccr2* etiolated seedlings.

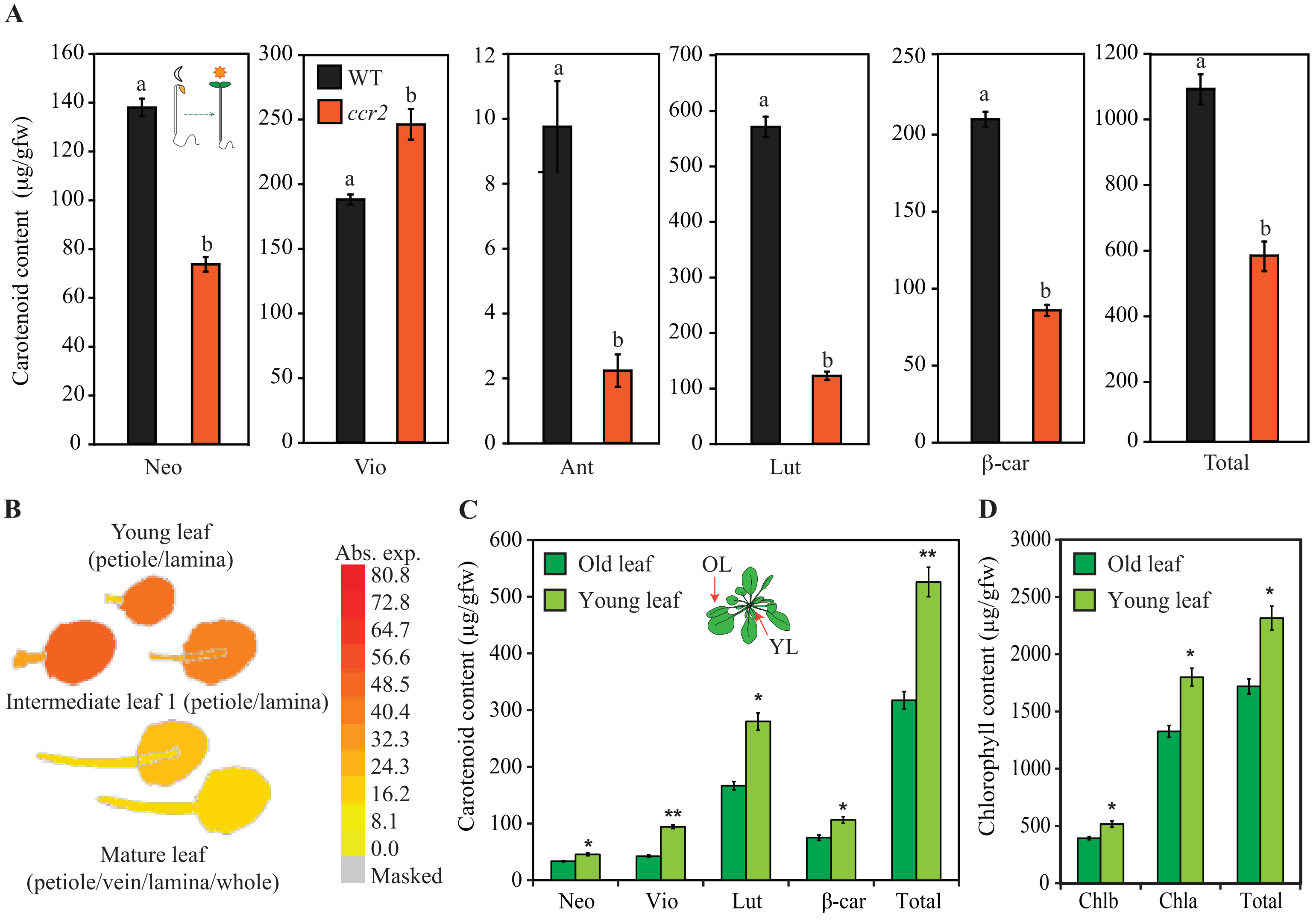

Figure S5. Pigment content and *εLCY* expression in young and old leaves

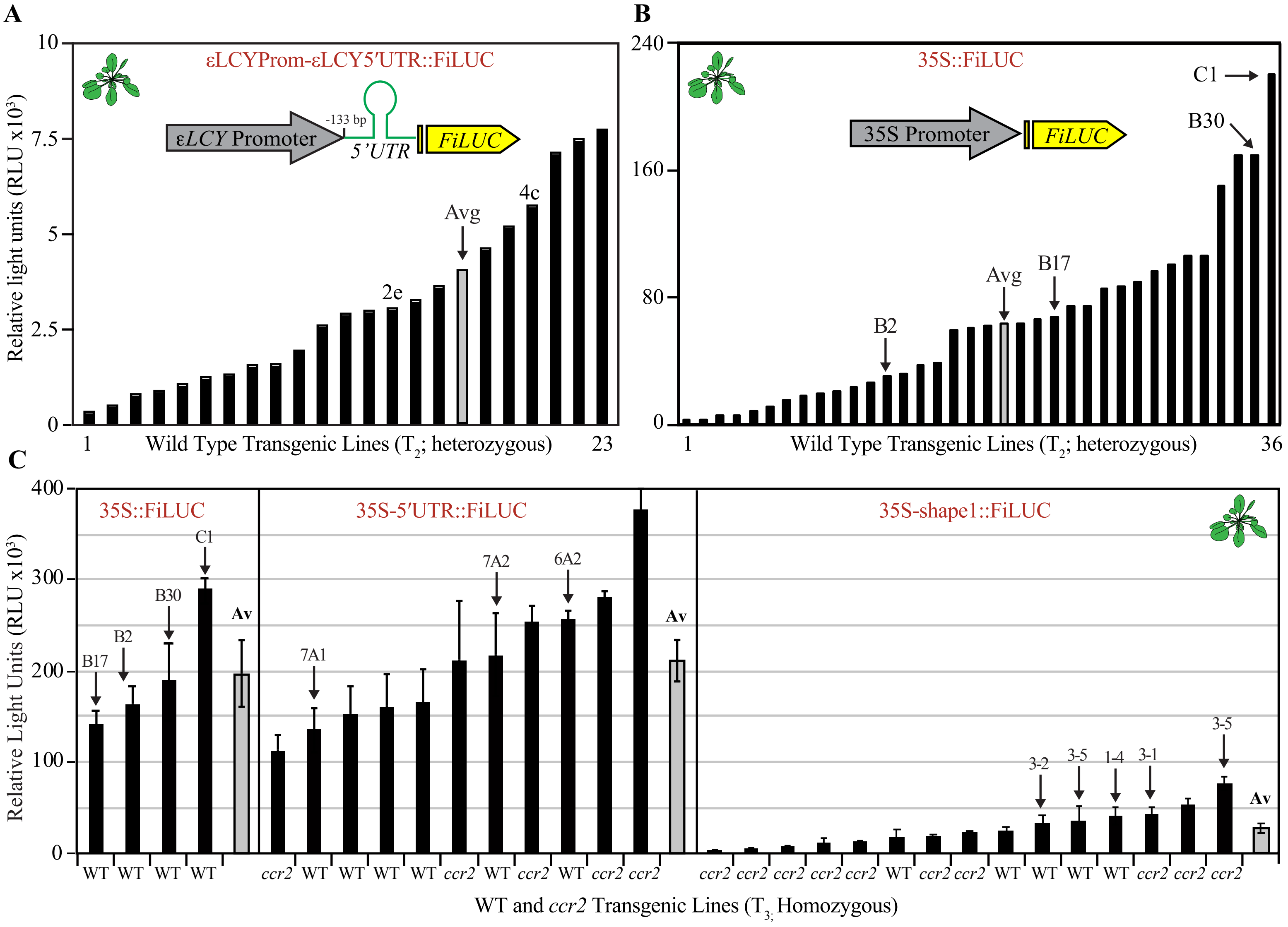

Figure S6. Luciferase activity and expression in plant tissues from independent transgenic lines harboring promoter-reporter gene fusions.

A Uncropped 5′RACE gel image

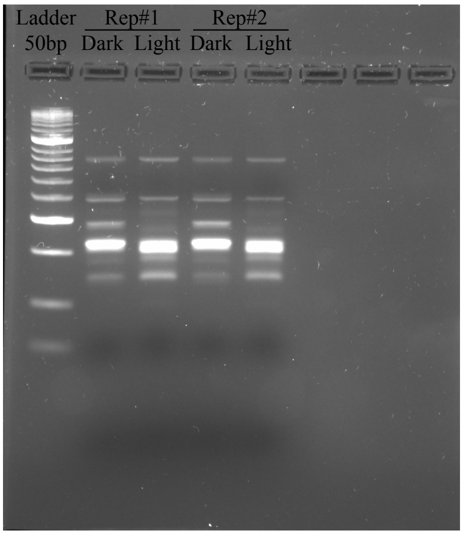

B *cis*-acting elements identified in the ε*LCY* promoter (-450 bp)

Motif	Position	Sequence	Function of cis-acting element
ABRE	-448	ACGTG	Abscisic acid responsive element (ABRE) - associated with ABA
CAAT box	-434	TCAAT	Signals binding site for the RNA transcription factor
	-234	CCAAT	
TATA box	-357	TTATA	Binds TATA-binding protein (TBP) during transcription initiation
	-305	TTTTAAAT	
DPE	-343	GGTCG	Downstream promoter element (DPE) functions with INR to bind TFIID (Transcription factor II D) protein in TATA-less promoters
	-256	GGACC	
Inr element	-289	CCAATCC	Initiator (Inr) directs RNA polymerase to bind TFIID during transcription initiation in TATA-less promoters
	-222	TTACAC	
	-153	TCATTTT	
TCT-motif	-279	TCTTAC	light-responsive
G-Box	-219	CACGTT	light-responsive
IME-like motif	-137	TTNGATYTG	Intron mediated enhancement (IME) - transcription enhancer
Pyrimidine patch	-179	TCCTCTC	component of core promoter associated with both the TATA-box and the Inr motif
	-86	CTCTTTCT	
	-37	TCTTCTC	
IRES Motif	-82	red font	Internal ribosome entry site (IRES) initiation protein synthesis by cap-independent mechanisms

C Position of *cis*-acting elements and conserved domains relative to 5′UTR within ε*LCY* promoter

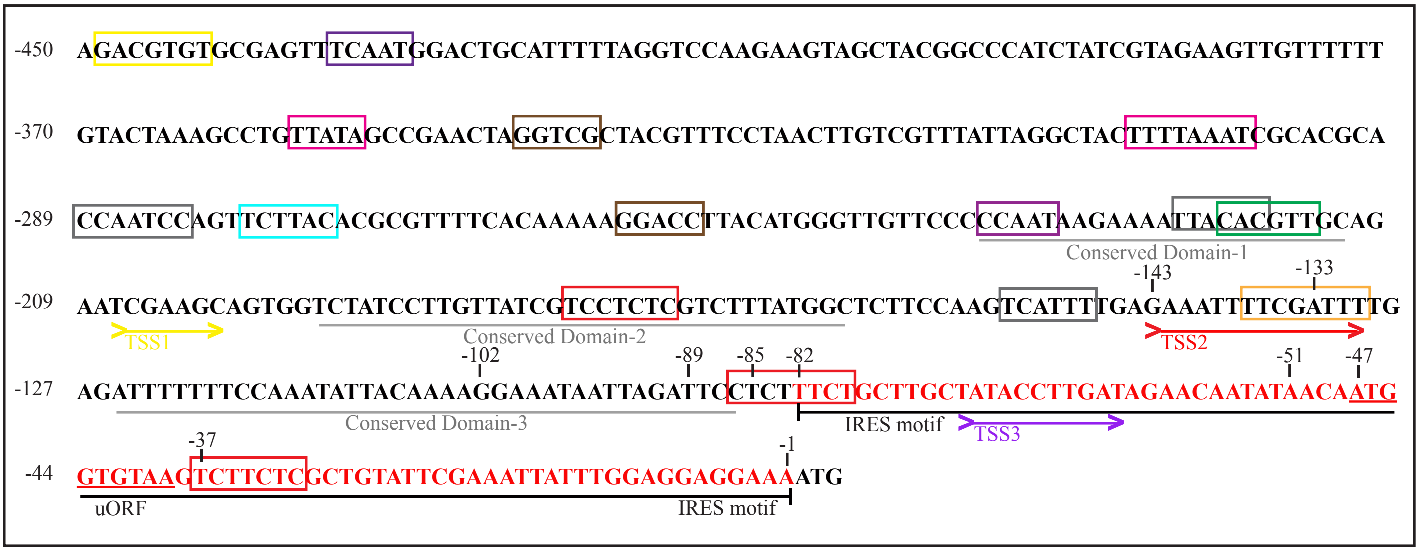

D Sequence alignment of ε*LCY* 5′UTR showing mutation and deletion (MutDel) fragments.

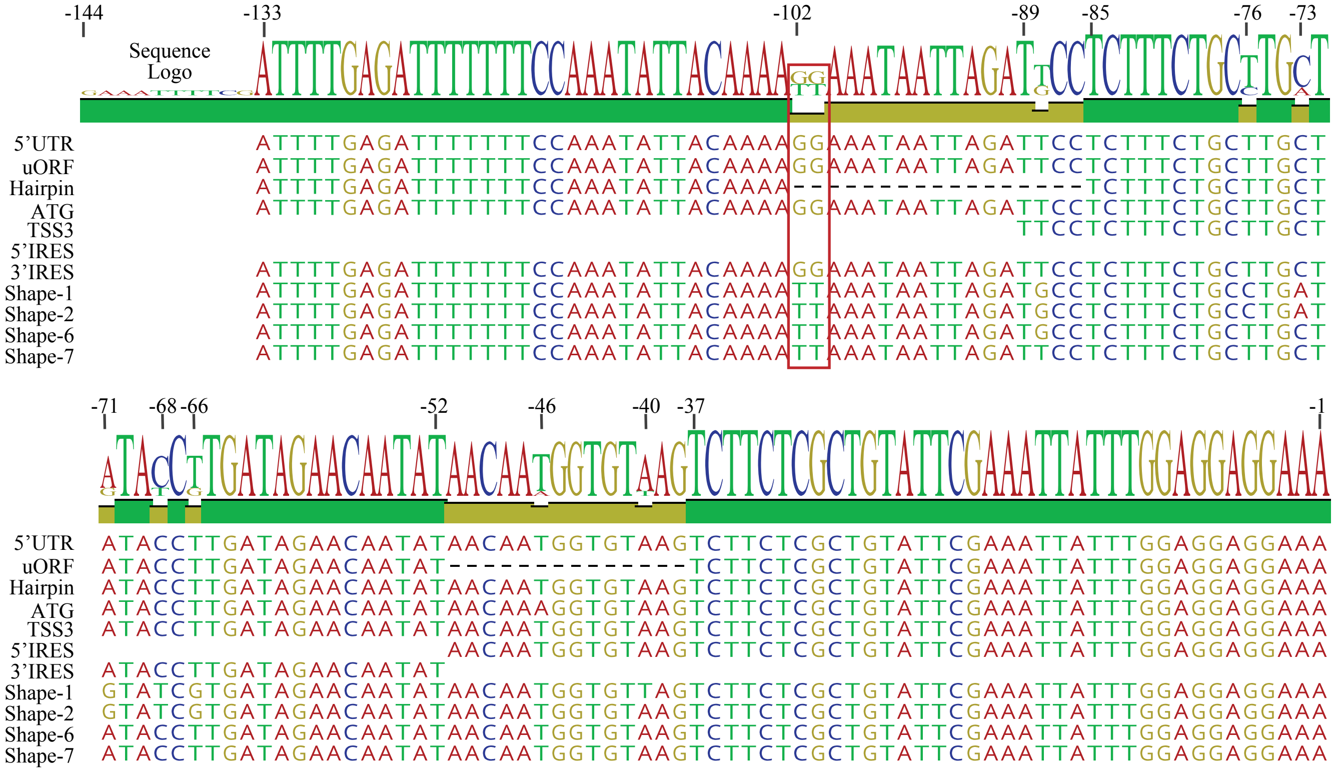

Figure S7. Deletions, mutations, cis-acting motifs and conserved domains in the ε*LCY* promoter and 5’UTR.

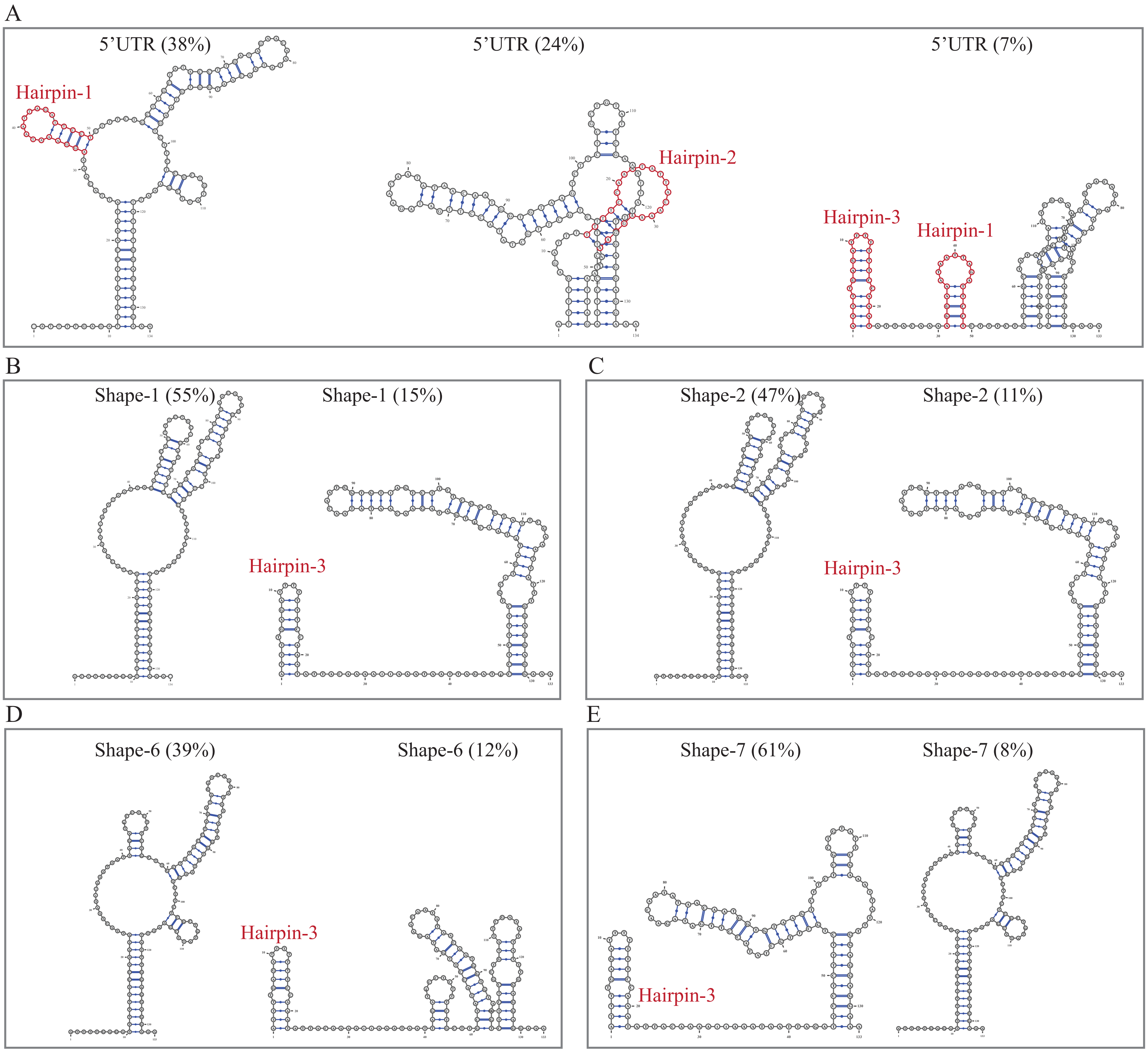

Figure S8. Representative RNA secondary structural plots and preduction probabilities.

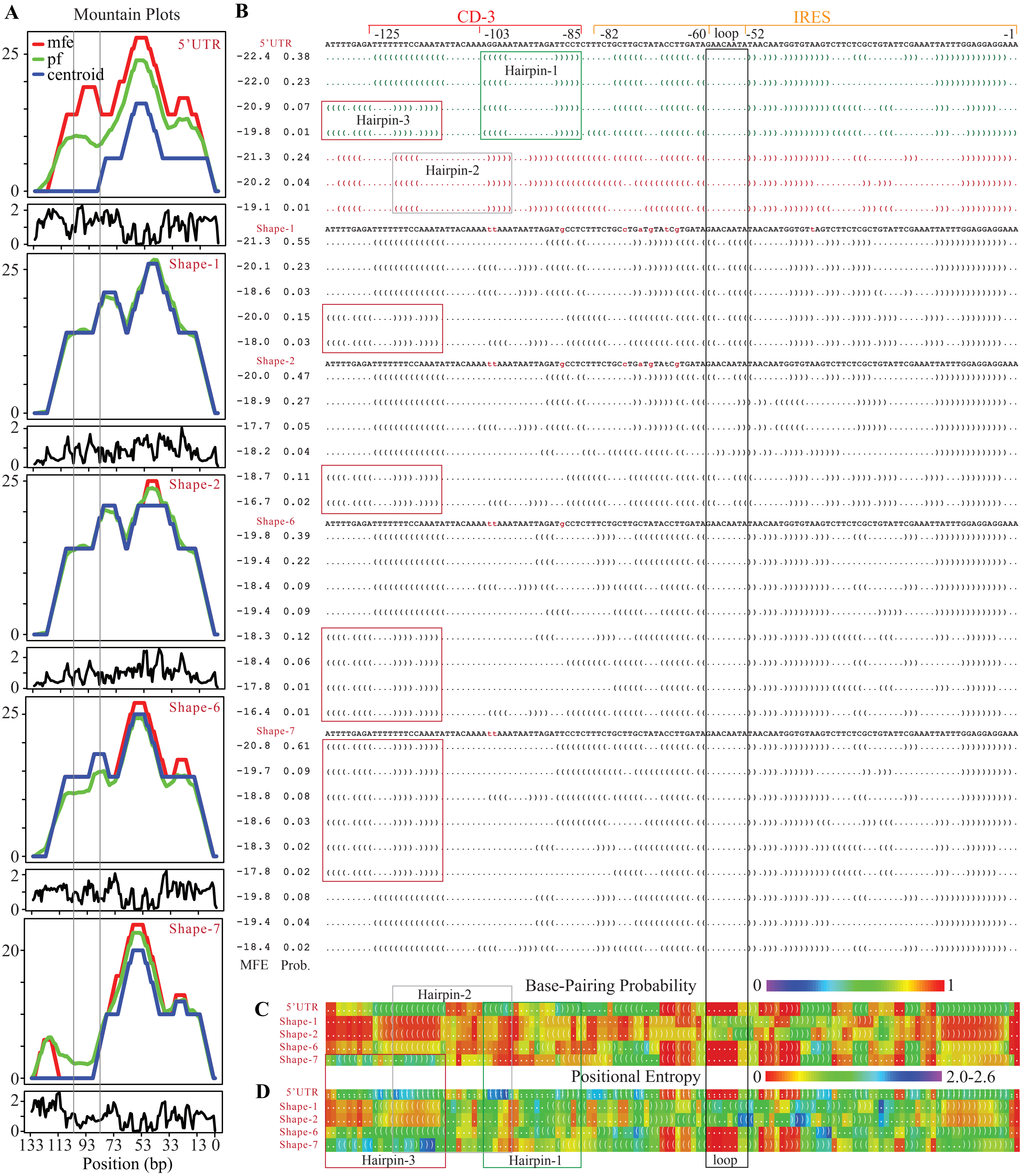

Figure S9. *In silico* RNA structural analysis of the *εLCY* shape variants modulating a posttranscriptional expression platform
