# Supplementary material for: Plastid-mediated feedback regulation of Arabidopsis LYCOPENE EPSILON CYCLASE is modulated by the promoter and a 5′UTR structural variant harbouring a conserved IRES
Source: BMC Biol. 2025 Nov 4;23:334. doi: 10.1186/s12915-025-02436-z (PMC12584516; doi:10.1186/s12915-025-02436-z)
Supplement: Supplementary file 2 — Additional file 2: Table S1. Correlations between the regulation of εLCY and carotenoid levels in plants. Table S2. Carotenoid percent composition in rosette leaf tissues from ccr2 lines harboring 35Senh::SSU-PaCrtI. Table S3. Primers used for qPCR, cloning, and 5'Rapid Amplification of cDNA Ends. [file 12915_2025_2436_MOESM2_ESM.pdf]

Table S1. Correlations between the regulation of *εLCY* and carotenoid levels in plants.

Species	Tissue	Genetic Locus Impaired	Exogenous Gene OE	β-car level	Carotenoids Accumulated (other than β-car)	Carotenoids Reduced (other than β-car)	*εLCY* transcription	Ref.
Tomato (*Delta*)	Ripe fruit	Del (*εLCY* allele)	-	∨	δ-car, α-car, Lut	Lyc	∧ in fruit	Ronen 1999
Tomato (*Ailsa Craig*)	Fruit	-	*CrtI*	∨ in mature green fruit, ∧ in ripe fruit	Ripe fruit: Neo, Vio; Leaves: Vio	Ripe and green fruits: All *cis*-car; Leaves: Total Chl, Lut, Neo	∧ in 3 days post breaker fruit	Enfissi et al 2017
Tomato (*tangerine*^{3183})	Fruit	*CRTISO*	*CrtI*	∨ in mature green fruit, ∧ in ripe fruit	Ripe fruit: Lyc, Lut, Neo, Vio; Leaves: Vio	Ripe and green fruits: All *cis*-car, Leaves; Lut, Neo	∧ in 3 days post breaker fruit	Enfissi et al 2017
Tomato (*old gold crimson*)	Fruit	*CYCB* (*βLCY*)	*CrtI*	∧ in ripe fruit	Ripe fruit: Lut	Ripe fruit: Phy, Phf	∧ in 3 days post breaker fruit	Enfissi et al 2017
Arabidopsis	Etiolated Seedlings	*CRTISO (ccr2)*		absent	*cis*-carotenes	All	∨	Cuttriss et al., 2007
Canola	Seeds	*εLCY* (RNAi)	-	∧	Zea, Vio, and Lut	N/A	∨ (RNAi)	Yu 2008
Sweet potato	Transgenic calli	*εLCY* (RNAi)	-	∧	All β-branch car, ABA	Lut	∨ (RNAi)	Kim 2013
Potato	Tubers	*εLCY* (Tuber specific RNAi)	-	∧	Zea, Ant, Vio and Neo,	N/A	∨ (RNAi)	Diretto 2006
Maize	Kernels	*εLCY*	-	∧	N/A	N/A	Mutated (Variable positions incl. 5'TE)	Harjes 2008
Maize	Kernels	*εLCY* & *crtRB1* (*BCH1*)	-	∧	N/A	N/A	Mutated (5'TE)	Muthusamy 2015
Maize	Kernels	*εLCY*	-	∧	N/A	N/A	Mutated (5'TE)	Zunjare 2018
Melon	Mature fruit	*CmOr*	-	∧	ζ-car	N/A	∨	Chayut et al., 2015
Tobacco	Leaves	*εLCY* (RNAi)	-	∧	Vio, Neo, Chl-b, Chl-a	N/A	∨ (RNAi)	Shi 2015
Sweet potato	Tubers	*εLCY* (RNAi)	-	∧	Zea, β-cry	α-car	∨ (RNAi)	Ke 2019
Wheat (ssp. durum)	Leaves	*LCYE-A1,* W437	-	∧	N/A	N/A	Mutated (TILLING)	Richaud 2018

Abbreviations: ABA, abscisic acid; Ant, Antheraxanthin; Chl-a, Chlorophyll-a; Chl-b, Chlorophyll-b; cis-car, cis-carotenoids; Lut, Lutein; Lyc, Lycopene; Neo, Neoxanthin; Overexpressed, OE; Phy, Phytoene; Phyf, Phytofluene; Ref, references; RNAi, RNA interference; TE, transposon Insertion; Vio, Violaxanthin; Zea, Zeaxanthin; α-car, Alpha-carotene; β-cry, β-cryptoxanthin; δ-car, Delta-carotene; ζ-Car, ζ- Carotene; "∨", decreased; "∧", increased; "N/A", not available/unknown.

Table S2. Carotenoid percent composition in rosette leaf tissues from *ccr2* lines harboring *35Senh*::*SSU-PaCrtI*

Germplasm	Line #	Chl a/b Ratio	Percentage Carotenoid Composition						Segregation Chi^2 p-value
			**Neoxanthin**	**Violaxanthin**	**Antheraxanthin**	**Lutein**	**Zeaxanthin**	**β-carotene**	
ccr2 PaCrtI	5	2.8	11.3	27.0	7.3	15.5	2.2	36.7	0.64
ccr2 PaCrtI	9	2.6	12.0	29.8	7.7	16.6	2.0	31.9	0.68
ccr2 PaCrtI	11	2.7	11.9	30.4	7.2	16.1	2.0	32.4	0.70
ccr2 PaCrtI	26	2.9	10.4	34.6	5.6	14.1	1.6	33.6	0.72
ccr2 PaCrtI	**Average**	**2.7**	**11.8**	**29.1**	**7.4**	**16.0**	**2.1**	**33.6**	
ccr2	**2.1**	**2.6**	**11.7**	**29.9**	**6.8**	**17.1**	**1.9**	**32.6**	

Chi^2 P-value >0.05 reveals lines that show 3:1 segregation of green to white seedlings.

Table S3. Primers used for qPCR, cloning, and 5'Rapid Amplification of cDNA Ends

	Primer Name	Gene Name	Accession	Sequnce (5' to 3')
qPCR	AtCycloProm-cc1F	Cyclophilin (Promoter)	AT2G29960	TCTCTGACCTTCTTTTGTTGCC
	AtCycloProm-cc1R	Cyclophilin (Promoter)	AT2G29960	TCCAATGCTAACATACGCTGG
	AtPP2A-cc1F	Protein Phosphotase 2A	AT1G13320	CTTCGTGCAGTATCGCTTCTC
	AtPP2A-cc1R	Protein Phosphotase 2A	AT1G13320	ATTGGAGAGCTTGATTTGCG
	AtLUC-cc1F	Firefly Luciferase	-	TGGAACCGCTGGAGAGCAAC
	AtLUC-cc2R	Firefly Luciferase	-	CGCAACTGCAACTCCGATAA
	AteLCY-cc1F	εLCY	AT5G57030	TCGGTTTGTGTAGTGTCAGAGC
	AteLCY-cc1R	εLCY	AT5G57030	AAGGCTAAACCAGCAGGACC
	AtLHCB2-F	Light Harvesting Complex	AT1G29930	CAGCTATCCAACAATCCTCCTTCG
	AtLHCB2-R	Light Harvesting Complex	AT1G29930	TTCTCCGAGAATGGTCCCAAGTAC
	AtRBCS1a-F	Rubisco Small Subunit	AT1G67090	TACCTTCCTGACCTTACCGATTC
	AtRBCS1a-R	Rubisco Small Subunit	AT1G67090	CGATGATCCTAATGAAGGCATTG
	TIP41-cc1F	TIP41-like family protein	AT4g34270	CAACGCCATACTGTGGAAGTG
	TIP41-cc1R	TIP41-like family protein	AT4g34270	AAATCGCAAGAGGAGGAACC
Cloning	M13F	pGEM-T		GTAAAACGACGGCCAGT
	M13R	pGEM-T		CACACAGGAAACAGCTATGACCATG
	HindIII_T35_F1	CaMV35s enhancer		AAGCTTCCATTGCCCAGC
	HindIII+XbaI_T35_R1	CaMV35s enhancer		AAGCTTtctagaGTCCTCTCCA
	1F_Mut5UTR			tctagaATTTTGAGATTTTTTTCC
	1R_Mut5UTR			ccatggTTTCCTCCTCCAAATAATTTCGAATACAGCGAGAAGACTTACACC
	4F_Mut5UTR			tctagaATTTTGAGATTTTTTTCCAAATATTACAAAAttAAATAATTAGATgCCTCTTTCTGCTTGCTATACCTT
	6F_Mut5UTR			tctagaATTTTGAGATTTTTTTCCAAATATTACAAAAttAAATAATTAGATTCCTCTTTCTGCTTGCTATACCTT
	eLCYProm-cc4F	εLCY Promoter (450 bp)	AT5G57030	GCTCTAGACGTGTGCGAGTTTCAATGG
	eLCYProm-cc1R	εLCY Promoter (450 bp)	AT5G57030	CATGCCATGGTTTCCTCCTCCAAATAATTTCG
	eLCY 5UTR-cc1R	eLCY 5'UTR 3'deletion (401 bp)	AT5G57030	CATGCCATGGTTATATTGTTCTATCAAGGTATAGC
	eLCY 5UTR-cc1F	eLCY 5'UTR (133 bp)	AT5G57030	CCCAAGCTTATTTTGAGATTTTTTTCCAAATATTAC
	35enh-cc1F	CaMV35s enhancer		CCCAAGCTTCCATTGCCCAGCTATCTGTC
	35enh-cc1R	CaMV35s enhancer		CCCAAGCTTGTCCTCTCCAAATGAAATGAAC
	NosTmod1-cc1F	Modified NosTerminator		CAAGATCTGCTCATGAATTTCCCCTAGGATCGTTCAAACATTTGGC
	NosTmod-cc1R	Modified NosTerminator		CAAGATCTGCAGGAATTAATTC
5'RACE	GeneRacer™ 5'			CGACTGGAGCACGAGGACACTGA
	GeneRacer™ 5' Nested			GGACACTGACATGGACTGAAGGAGTA
	eLCy-cc2R			CAGCGAAATCTTCTCTCACC
	eLCY-5'RACE R-primer1 (Nested)			ACCGCCATTGCTGCGAAATTCCT
	PDS3-5'RACE R-primer1			TCGCCGCAGAAACATTCCCAAAC
	PDS3-5'RACE R-primer2(Nested)			CCGCAGAAACATTCCCAAACACAACC
	ZDS-5'RACE R-primer1			GGCTCTGGAGGAAATAACCCCTTTGG
	ZDS-5'RACE R-primer2 (Nested)			AACCCCTTTGGAGCGTTAACACTC
	ZISO-5'RACE R-primer1			GGTCGAGTTTCCGAGGCGAGGAT
	ZISO-5'RACE R-primer2 Nested			CGAGGCGAGGATGAGCTGGGATAC
	CRTISO-5'RACE R-primer1			CCAGAACCGATAACAATGGCGTCGT
	CRTISO-5'RACE R-primer2 (Nested)			GGCGTCGTACAGACTCTCTCCTCCA
	bLCY-5'RACE R-primer1			GCCCTTTTCTTCACACCAAGCCTAACC
	bLCY-5'RACE R-primer2 (Nested)			TCGAGCTTGTTGGGTGTTTTCAACAG
	DXS-5'RACE R-primer1			TGTTGTTGGGTTTCAGACATGGTGATGG
	DXS-5'RACE R-primer2 (Nested)			TCAGACATGGTGATGGAAGATCTGTAAC
	PTOX-5'RACE R-primer1			GAGCTGTACGAAACGGCGGCTCT
	PTOX-5'RACE R-primer2 (Nested)			ACGGCGGCTCTAGAGCGTCGAAG
	DXR-5'RACE R-primer1			CCTCTCCCTTGATTCCTCCTCCTCA
	DXR-5'RACE R-primer2 (Nested)			CCCACCTGAGAGTTTAGGGATTGGA
	HDR-5'RACE R-primer1			GCCAGACGAGCACCGGACTGATA
	HDR-5'RACE R-primer2 (Nested)			AATCGGCTGAATTGGAGCGCAAC
	PSY-5'RACE R-primer1			GCGCAAACTTCACCGCATCG
	PSY-5'RACE R-primer2			CCCACAATTGTTCATTGGGTCTGG
